# Hypothalamic Glutamate/GABA Cotransmission Modulates Hippocampal Circuits and Supports Long-Term Potentiation

**DOI:** 10.1523/JNEUROSCI.0410-21.2021

**Published:** 2021-09-29

**Authors:** Musa Iyiola Ajibola, Jei-Wei Wu, Wahab Imam Abdulmajeed, Cheng-Chang Lien

**Affiliations:** ^1^Taiwan International Graduate Program in Interdisciplinary Neuroscience, College of Life Sciences, National Yang Ming Chiao Tung University and Academia Sinica, Taipei, 115, Taiwan; ^2^Institute of Neuroscience, College of Life Sciences, National Yang Ming Chiao Tung University, Taipei, 112, Taiwan; ^3^Brain Research Center, National Yang Ming Chiao Tung University, Taipei, 112, Taiwan; ^4^Department of Physiology, Faculty of Basic Medical Sciences, College of Health Sciences, University of Ilorin, Ilorin, 24003, Nigeria

**Keywords:** cotransmission, GABA, glutamate, hypothalamus, long-term potentiation, supramammillary nucleus

## Abstract

Subcortical input engages in cortico-hippocampal information processing. Neurons of the hypothalamic supramammillary nucleus (SuM) innervate the dentate gyrus (DG) by coreleasing two contrasting fast neurotransmitters, glutamate and GABA, and thereby support spatial navigation and contextual memory. However, the synaptic mechanisms by which SuM neurons regulate the DG activity and synaptic plasticity are not well understood. The DG comprises excitatory granule cells (GCs) as well as inhibitory interneurons (INs). Combining optogenetic, electrophysiological, and pharmacological approaches, we demonstrate that the SuM input differentially regulates the activities of different DG neurons in mice of either sex via distinct synaptic mechanisms. Although SuM activation results in synaptic excitation and inhibition in all postsynaptic cells, the ratio of these two components is variable and cell type-dependent. Specifically, dendrite-targeting INs receive predominantly synaptic excitation, whereas soma-targeting INs and GCs receive primarily synaptic inhibition. Although SuM excitation alone is insufficient to excite GCs, it enhances the GC spiking precision and reduces the latencies in response to excitatory drives. Furthermore, SuM excitation enhances the GC spiking in response to the cortical input, thereby promoting induction of long-term potentiation at cortical-GC synapses. Collectively, these findings provide physiological significance of the cotransmission of glutamate/GABA by SuM neurons in the DG network.

**SIGNIFICANCE STATEMENT** The cortical-hippocampal pathways transfer mnemonic information during memory acquisition and retrieval, whereas subcortical input engages in modulation of communication between the cortex and hippocampus. The supramammillary nucleus (SuM) neurons of the hypothalamus innervate the dentate gyrus (DG) by coreleasing glutamate and GABA onto granule cells (GCs) and interneurons and support memories. However, how the SuM input regulates the activity of various DG cell types and thereby contributes to synaptic plasticity remains unexplored. Combining optogenetic and electrophysiological approaches, we demonstrate that the SuM input differentially regulates DG cell dynamics and consequently enhances GC excitability as well as synaptic plasticity at cortical input-GC synapses. Our findings highlight a significant role of glutamate/GABA cotransmission in regulating the input-output dynamics of DG circuits.

## Introduction

The cortical-hippocampal pathways transfer mnemonic information during memory acquisition and retrieval and play a central role in spatial navigation, declarative memory, and complex information processing (Morris et al., 1982; Squire, 1992; Henze et al., 2002; Amaral et al., 2007; Nakashiba et al., 2012; Buzsáki and Moser, 2013; Ito et al., 2018; Hainmueller and Bartos, 2020). The synapses present along this pathway have been characterized extensively as substrates for distinct types of memories (O'Keefe and Dostrovsky, 1971; Nakazawa et al., 2002; Remondes and Schuman, 2004; Kitamura et al., 2015). The granule cells (GCs), the principal cells of the dentate gyrus (DG), receive cortical inputs (Buckmaster et al., 1996; Scharfman and Myers, 2012; Zhang et al., 2013) and segregate them into distinct neural codes after integration (Yassa and Stark, 2011; Fernández-Ruiz et al., 2021). In addition, subcortical inputs from different areas of the brain innervate GCs (Nyakas et al., 1987; Nitsch and Leranth, 1994; Unal et al., 2015). However, information regarding the synaptic organization and functions of the subcortical inputs is relatively limited.

The supramammillary nucleus (SuM) in the hypothalamus consist of subsets of neurons that innervate the DG and the CA2/CA3 subfields (Borhegyi and Leranth, 1997; Chen et al., 2020; Kohara et al., 2014; Leranth and Hajszan, 2007; Magloczky et al., 1994; Nitsch and Leranth, 1994; Pan and McNaughton, 2004; Vertes, 2015). SuM-DG connections are known to regulate hippocampal theta oscillations (Ito et al., 2018; Kocsis and Kaminski, 2006; Kocsis and Vertes, 1994; Ruan et al., 2011; Thinschmidt et al., 1995), learning (Aranda et al., 2008; Hernandez-Perez et al., 2015; Ruan et al., 2011; Shahidi et al., 2004), rapid eye movement sleep (Renouard et al., 2015), arousal (Pedersen et al., 2017), and explorative locomotor activities (Ito et al., 2009; Sławińska and Kasicki, 1998; Wirtshafter et al., 1998). Moreover, the SuM synchronizes with the DG in the regulation of goal-directed behavior during spatial navigation (Ito et al., 2018; Li et al., 2020). Recently, a subset of SuM neurons was reported to signal contextual information to the DG (Chen et al., 2020).

SuM neurons form synapses with the perisomatic region of the GC, and their axonal terminals coexpress the vesicular glutamate transporter Type II (VGluT2) and vesicular GABA transporter (VGAT) (Boulland et al., 2009; Soussi et al., 2010; Root et al., 2018). Notably, VGluT2 and VGAT are segregated to distinct synaptic vesicles at the SuM terminals in the DG (Boulland et al., 2009; Root et al., 2018). The segregated localization of neurotransmitter vesicles in the same terminals suggests differential cotransmission of glutamate and GABA at the SuM-DG synapses (Dugué et al., 2005; Somogyi, 2006; Vaaga et al., 2014). Consistent with this observation, SuM terminals in the DG simultaneously release both glutamate and GABA to GCs and GABAergic interneurons (INs) (Pedersen et al., 2017; Hashimotodani et al., 2018; Billwiller et al., 2020). Of note, the ratio of the glutamate- and GABA-mediated components recorded in INs varied from 0.34 to 7.7 (Hashimotodani et al., 2018). Given the diverse types of INs present in the DG, glutamate and GABA are likely to be differentially cotransmitted in an IN subtype-specific manner (Hájos et al., 1996; Hosp et al., 2014; Liu et al., 2014; Hsu et al., 2016; Booker and Vida, 2018). Differential recruitment of distinct IN subtypes can powerfully modulate the input and output logic of DG (Miles et al., 1996; Lee et al., 2016). However, whether the SuM input differentially recruits distinct IN subtypes in the DG remains unknown. Moreover, GABA, which is cotransmitted with glutamate by the SuM, is known to exert shunting inhibitory effects on GCs and thereby could bidirectionally control action potential firing in GCs (Chiang et al., 2012; Heigele et al., 2016). Yet it is unclear how the SuM input regulates the input-output dynamics of DG circuits.

Here, combining electrophysiological and optogenetic approaches, we demonstrate that SuM input differentially regulates the activity of DG neurons. Optogenetic activation of SuM input was able to excite dendrite-targeting INs (D-INs), but was not sufficient to activate soma-targeting INs (S-INs) and GCs. Consistent with these observations, GCs and S-INs received predominantly synaptic inhibition, whereas, D-INs received predominantly synaptic excitation. As a consequence, activation of the SuM input enhances the temporal precision of GC firing and shortened spike latencies in D-INs. Moreover, coactivation of the SuM input with the cortical input enhanced the responses of GCs to the cortical input. Finally, repeated coactivation of the SuM and cortical inputs resulted in enhanced LTP at the cortical-GC synapses.

## Materials and Methods

### 

#### 

##### Animals

We used the VGluT2-Cre driver line (*Slc17a6^tm2(cre)Lowl^*/J, stock #016963), VGAT-Cre driver line (*Slc32al^tm2(cre)Lowl^*/J, stock #028862), and Gad2-Cre driver line (*Gad2^tm2(cre)Zjh^*/J, stock #010802) obtained from The Jackson Laboratory, and WT mice with C57BL/6J genetic background obtained from National Laboratory Animal Center. Both male and female mice (3-5 months old) were used for the electrophysiological experiments. The mice were housed in a room with a reverse 12 h light/12 h dark cycle and were provided with food and water *ad libitum*. The protocols and procedures for the animal experiments were in accordance with the national and institutional guidelines and were approved by the Animal Care and Use Committee of National Yang Ming Chiao Tung University.

##### Viruses

For the optogenetic experiments, we virally expressed channelrhodopsin (ChR2)-eYFP on SuM neurons by injecting an adeno-associated virus (AAV) serotype 5-CaMKIIα-ChR2(H134R)-eYFP (4.1 × 10^12^ vector genomes/ml, University of North Carolina) into the SuM of WT mice. To target glutamatergic and GABAergic neurons in the SuM selectively, an AAV5 vector carrying a Cre-inducible ChR2-eYFP transgene (AAV5-EF1α-DIO-hChR2-(H134R)-eYFP) (4.3 × 10^12^ vector genomes/ml, University of North Carolina) was injected into the SuM of VGluT2-Cre, VGAT-Cre, and Gad2-Cre mice.

##### Stereotaxic injection

For the retrograde tracer and virus injections, the mice were anesthetized with 4% isoflurane (v/v; Halocarbon Laboratories) in a 100% oxygen-containing induction chamber. The scalp was shaved, and the mice were transferred to a stereotaxic frame (IVM-3000; Scientifica) for the surgery. The mouth and nose of each mouse were covered using an anesthetizing mask that was supplied with ∼1.5% isoflurane and had an airflow rate of 4 ml/min. To maintain the body temperature of the mice at 34°C-36°C, a biological temperature controller pad (Physitemp Instruments, or TMP-5b, Supertech Instruments) remained placed under the body of each mouse throughout the surgical procedure. The head was fixed using two ear bars; 75% ethanol was applied to the scalp to sterilize the surgical area, and an ophthalmic gel was applied to the eyes to avoid dryness. An analgesic (ketorolac, 6 mg/kg) was administered intraperitoneally. For the delivery of the tracer, unilateral or bilateral craniotomy was performed at the AP and ML coordinates of the dorsal DG (AP: −1.80 mm, ML: ±1.30 mm). Then the tracer was delivered into the DG at the DV coordinate (DV: −2.20 and −2.0 mm). To target the SuM neurons, unilateral or bilateral craniotomy was performed over the SuM (AP: −2.85 mm, ML: ±0.15 mm). Then viral vectors were delivered into the SuM at DV, −4.86 mm. The viral vectors (0.2-0.4 µl) and red retrobeads (0.2 µl) (LumaFlour) were delivered to the SuM and DG, respectively, using a 10 µl NanoFil syringe (World Precision Instruments) and a 34-G beveled metal needle. The injection volume (0.2-0.4 µl) and flow rate (0.1 µl/min) were controlled using a nanopump controller (KD Scientific). Subsequently, the needle was raised 0.1 mm above the site of injection for an additional 10 min to minimize the upward flow of the viral solution. Finally, the needle was gradually withdrawn. After the injection was performed, the incision was sutured, and the mice were transferred to the cage for recovery.

##### Preparation of brain slices

Acute brain slices containing the hippocampal and SuM sections were prepared 1 week after the retrograde tracer injection or at least 3 weeks after the viral injection. Transverse brain slices were used for whole-cell patch-clamp recording of the DG neurons, while coronal brain slices were used for recording of retrobead-positive SuM neurons. The mice were anesthetized using isoflurane and decapitated rapidly. The brains were quickly removed and transferred to an ice-cold oxygenated (95% O_2_ and 5% CO_2_) sucrose solution containing the following (in mm): 87 NaCl, 25 NaHCO_3_, 1.25 NaH_2_PO_4_, 2.5 KCl, 10 glucose, 75 sucrose, 0.5 CaCl_2_, and 7 MgCl_2_. Next, 300-µm-thick slices were cut using a vibratome (DTK-1000; Dosaka). After sectioning, the slices were recovered at 34°C for 25 min in a holding chamber filled with an oxygenated sucrose solution, then transferred to room temperature (25 ± 2°C) for additional experiments.

##### Electrophysiology and optical stimulation

For the recordings, individual slices were transferred to a submerged chamber and were continuously perfused with oxygenated ACSF containing the following (in mm): 125 NaCl, 25 NaHCO_3_, 1.25 NaH_2_PO_4_, 2.5 KCl, 25 glucose, 2 CaCl_2_, and 1 MgCl_2_. The ChR2-eYFP expression pattern was confirmed using fluorescence, and the neurons in the DG were selected visually for recording under an infrared differential interference contrast microscope (IR-DIC, BX51WI, Olympus). The axonal terminals that expressed ChR2 were stimulated with 470 nm light transmitted through the objective from an LED source (LED4D162, driven by DC4104, Thorlabs).

Whole-cell patch-clamp recordings were performed using a Multiclamp 700B amplifier (Molecular Devices). The recording electrode pipettes (4-7 mΩ) pulled from borosilicate glass tubing (outer diameter, 1.5 mm; inner diameter, 0.86 mm; Harvard Apparatus) were filled with a high Cl^–^ internal solution, containing the following (in mm): 15 K-gluconate, 140 KCl, 0.1 EGTA, 2 MgCl_2_, 4 Na_2_ATP, 10 HEPES, 0.5 Na_3_GTP, and 0.4% biocytin (w/v, Invitrogen). In certain set experiments for the determination of spike-timing precision and spike phase, a low Cl^–^ internal solution containing the following (in mm): 136.8 K-gluconate, 7.2 KCl, 0.2 EGTA, 4 MgATP, 10 HEPES, 0.5 Na_3_GTP, 7 Na_2_-phosphoreatine (pH 7.3 with KOH) and 0.4% biocytin was used. The pipette capacitance was compensated in the cell-attached mode. To measure the EPSC and the IPSC, whole-cell recording was performed using a high Cl^–^ internal solution (E_GABA_ = ∼0 mV, E_AMPA_ = ∼0 mV), and the EPSCs and IPSCs were isolated using a pharmacological approach. Bath application of SR95531 (1 µm) and CGP55845 (1 µm) was used to block GABA_A_ and GABA_B_ receptors, respectively, while an ionotropic glutamate receptor blocker, kynurenic acid (Kyn, 2 mm), was used to block ionotropic glutamatergic transmission. The GABAergic component (IPSC) traces were obtained by digital subtraction of traces recorded after bath application of SR, CGP from the baseline traces recorded in the presence of ACSF. The glutamatergic component (EPSC) traces were obtained by digital subtraction of traces recorded in the presence of SR, CGP, and Kyn from the traces recorded in the presence of SR and CGP.

Cell-attached recording was performed with patch pipettes filled with a high Cl^–^ internal solution before whole-cell recording of current spikes in GCs and INs. A 5 Hz, 5 ms light pulse was applied with a 15 s intersweep interval, and 6 sweeps were recorded. The spike probability was determined as the percentage of spikes among 6 sweeps. In the dual recording experiments, the distance between the recorded pair was <200 µm. Although the serial resistance was not compensated, it was monitored continuously during the recording process. The recordings with the serial resistance < 25 mΩ were analyzed. Fast-spiking phenotype of hippocampal INs or putative S-INs recorded at room temperature (21°C-24°C) were defined by their maximal firing rate > 65 Hz and coefficient of variation of < 0.2 in response to 1 s depolarizing current injection (Lien and Jonas, 2003). The coefficient of variation was determined from the spike train with the maximal firing rate. For local field potential (LFP) recordings, a monopolar electrode (tip diameter; ∼10 µm) filled with ACSF was placed in the subiculum to stimulate the perforant path (PP) fibers. Trains of current pulses (10-500 µA, 0.1 ms) were applied every 15 s using a stimulus isolator (Isoflex, A.M.P.I.). The recording electrode (tip diameter, ∼5 µm) filled with ACSF was placed in the granule cell layer (GCL) to monitor the population spike (pSpike) in response to PP stimulation. Additional experiments were performed at stimulus intensities that evoked 30%-50% of the maximum pSpike amplitude and paired with the 10 ms light pulse for activation of the SuM input.

For the spike-timing precision experiments, sinusoidal waveforms were created and customized using Clampfit 10.3 (Molecular Devices). To test the ability of the SuM input to enhance spike-timing precision and phase, theta frequency (5 Hz trains of 5 pulses) sinusoidal current pulses were delivered into the GCs and were paired with 5 Hz square photostimulation of the SuM input. The 5 ms photostimulation was delivered during the ascending phase (31°-39°) of the sinusoidal waveform. The current injected (peak to trough, 50-150 pA) was set to evoke a single action potential close to the peak of the sinusoidal waveform while the membrane potential of the GCs was held at ∼−80 mV. Twenty sweeps were recorded at 15 s interval and superimposed to observe the precision of action potential generation. To determine the spike jitter and phase, the time point for the peak in each spike was converted to phase (angle) using the customized Python codes. The mean and the SD represented spike phase (latency) and spike jitter, respectively. All cells used for spike-timing precision experiments reliably generated EPSP in response to 5 Hz photostimulation of the SuM input. The signals were recorded using Multiclamp 700B amplifiers (Molecular Devices), filtered at 4 kHz, and sampled at 10 kHz using a digitizer (Digidata 1440A, Molecular Devices), which was controlled using pCLAMP version 10.3 (Molecular Devices).

##### *Post hoc* recovery and reconstruction of recorded neurons

To identify the recorded neurons (filled with 0.4% biocytin), brain slices were fixed overnight with 4% PFA (w/v) in PBS. After rinsing with PBS 3 times, 0.3% Triton X-100 (v/v; USB) in PBS (PBST) was added for 30 min, then blocked with 0.3% PBST and 10% normal goat serum (NGS, S-1000, Vector Laboratories) for 2 h. Slices were incubated with streptavidin-conjugated AlexaFluor-594 or -555 or -488 (1:400; Invitrogen) in 0.3% PBST and 5% NGS at 4°C overnight or 2 h at room temperature. After rinsing 6 times with PBS, slices were mounted onto slides with mounting medium Vectashield with DAPI (H-1200, Vector Laboratories). Confocal image stacks were reconstructed with Neuromantic 1.6.5 software (developed by Darren Myatt, University of Reading).

##### Immunohistochemistry

WT mice (3 months old) with AAV5-CaMKIIα-ChR2-eYFP injected into the SuM were deeply anesthetized using isoflurane and perfused transcardially with 20 ml of ice-cold PBS, followed by 50 ml of 4% PFA. The fixed brain specimens were excised and postfixed in 4% PFA for an additional 6 h or overnight. Next, dehydration was performed by incubation in 15% sucrose for 4 h, followed by 30% sucrose in PBS for 2 h. The brain specimens were sectioned coronally into 50 µm slices using a microtome (SM2010R, Leica Microsystems). The brain slices were rinsed with PBS 3 times and blocked by treating with 0.3% PBST and 5% NGS for 2 h. The slices were then incubated in a cocktail of rabbit anti-GFP antibody (1:1000, Abcam, ab290), rabbit anti-VGluT2 antibody (1:500, VGluT2-135 403, Synaptic System), and mouse anti-VGAT antibody (1:250, VGAT-131 011; Synaptic System) at 4°C for 24 h.

Next, the slices were rinsed 3 times with PBS and incubated in cocktails of fluorescent secondary antibodies, AlexaFluor-488 anti-rabbit, AlexaFluor-594 anti-rabbit, and AlexaFluor-647 anti-mouse at room temperature for 2 h or overnight at 4°C. The procedures were performed under continuous shaking conditions. After rinsing 6 times with PBS, the sections were mounted using the mounting medium Vectashield with DAPI. Fluorescent images were taken using a confocal microscope (Leica SP5 module, Leica Microsystems) or (LSM 700, Carl Zeiss) using 20×, 40×, or 63× objectives and analyzed using ImageJ (National Institutes of Health, 1.52t). Single-plane coronal sections with bead expression were imaged using a Research High-Class Stereo Microscope System (SZX16, Olympus). For colocalization analysis of ChR2-eYFP-expressing boutons with VGluT2 and VGAT, boutons along ChR2-eYFP expressing axons were identified in *z*-stack images, examined for colocalization, and counted using cell counter plugin in Fiji (a distribution of ImageJ software, National Institutes of Health, 1.53c) (Billwiller et al., 2020).

##### Data analysis and statistics

Data were analyzed using Clampfit 10.3 (Molecular Devices), Prism 6.0 (GraphPad Software), or customized Python codes. The synaptic latency was determined as the time elapsed from the light onset to the onset of the synaptic response (Hsu et al., 2016). The onset of the synaptic response was determined by the intersection of a line through the 20% and 80% points of the rising phase of the EPSC or IPSC and the baseline. To calibrate evoked IPSCs during successive 5 Hz photostimulation, the EPSC obtained after bath application of SR95531 (1 µm) and CGP55845 (1 µm) was digitally subtracted from the mixed postsynaptic current (baseline). To calculate the conductance, the EPSC and the IPSC amplitudes were divided by their respective driving forces. The input resistance was determined by the ratio of a steady-state (the last 100 ms of a 1 s pulse) voltage response versus the injected 1 s hyperpolarizing (10 pA) current pulse (Liu et al., 2014). The magnitude of LTP was calculated 30-40 min after LTP induction. Data are presented as mean ± SEM. Error bars in figures also show SEMs. Statistical significance was tested using the unpaired *t* test, Mann–Whitney test, Wilcoxon signed-rank test, or two-way repeated-measures ANOVA followed by Bonferroni's *post hoc* tests.

## Results

### Anatomical and physiological features of DG-projecting SuM neurons

**Figure 1.**
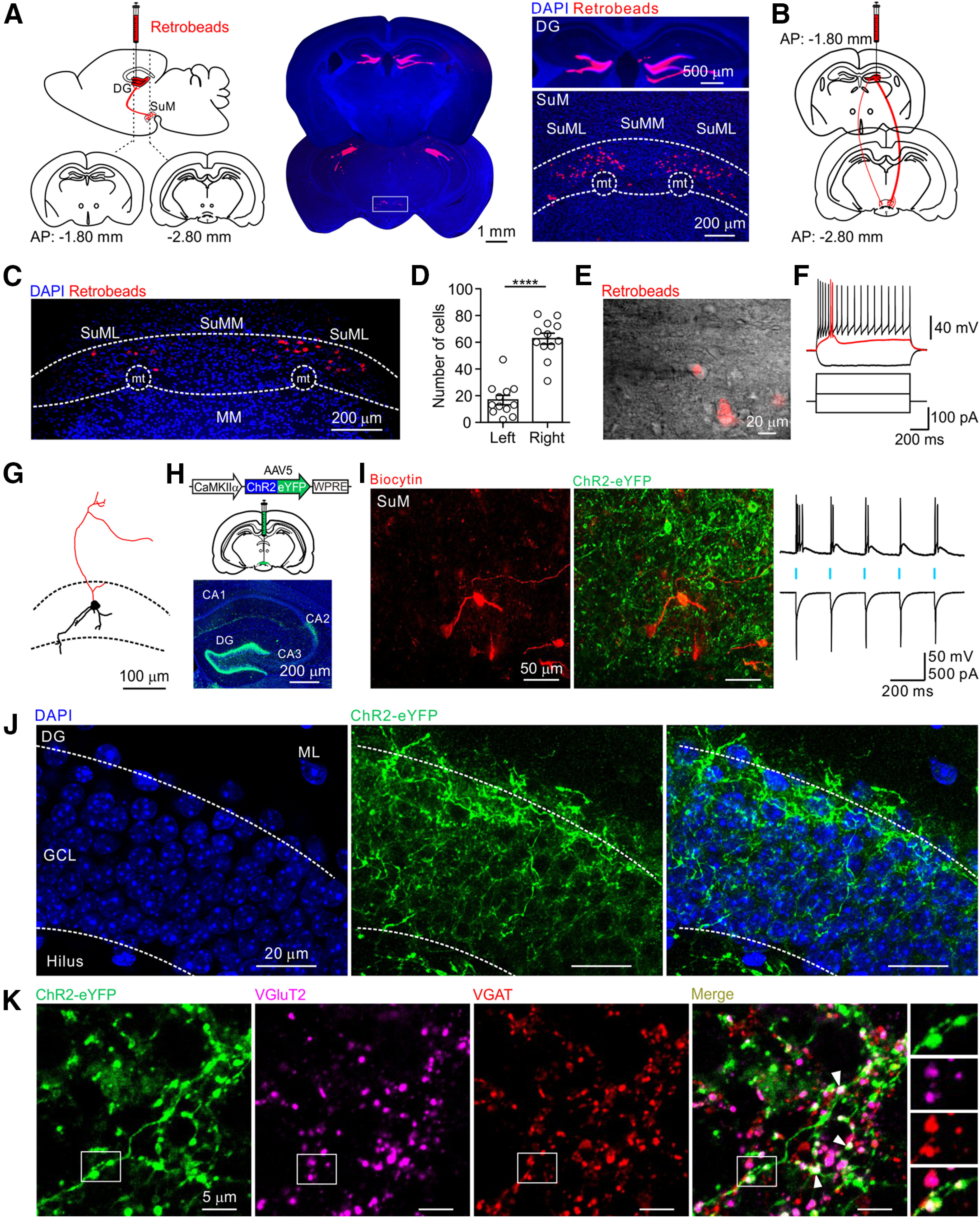
Anatomical and physiological characterization of DG-projecting SuM neurons. ***A***, Left, Schematic showing the location of retrogradely labeled cells in the SuM after bilateral red retrobead injections into the DG. Middle, Representative images of injection sites along the AP axis of the DG. Right top, High-magnification image of injection sites in the DG. Right bottom, Retrogradely labeled DG-projecting SuM neurons in the SuM area. ***B***, Schematic of unilateral red retrobead injection into the DG. ***C***, Retrogradely labeled DG-projecting SuM neurons were mainly located in the right SuML ipsilateral to the injection site. ***D***, Quantification of retrogradely labeled DG-projecting SuM neurons in the right and left SuML. Right SuML, 63 ± 4.2 cells; left SuML, 17 ± 3.5 cells; 12 slices from 3 mice; *p* < 0.0001, *U* = 2.0; Mann–Whitney test. ***E***, IR-DIC image showing whole-cell recording from bead-positive neuron in the SuM. ***F***, Representative firing pattern of a DG-projecting SuM neuron in response to 1 s current injection steps. ***G***, Morphologic reconstruction of a DG-projecting SuM neuron. Black represents soma and dendrites. Red represents axons. Black dotted lines indicate boundary of the SuM area. ***H***, Top, schematic of injection of AAV5-CaMKIIα-hChR2-eYFP into the SuM. Bottom, Representative confocal image stacks of coronal section depicting ChR2-eYFP expression in the DG and CA2. ***I***, Left, Biocytin-filled recording from a ChR2-expressing SuM neuron. Right, Traces of light-evoked spikes recorded from the same cell in the presence of Kyn (2 mm), in current clamp at −70 mV (top) and ChR2-mediated photocurrent recorded at ∼−70 mV in voltage clamp (bottom). Blue bars represent the light pulses (5 ms, 470 nm, 5 Hz light pulse). ***J***, Confocal image stacks of coronal section through the DG showing the projection pattern of SuM terminals in the DG. DAPI (left), ChR2-expressing SuM terminals (middle), and merged image (right). ***K***, Confocal image stacks of SuM axon terminals expressing ChR2-eYFP, VGluT2, and VGAT immunofluorescence and the merge image showing their colocalization on the labeled SuM terminals. Right, Putative boutons in the box. ****p < 0.0001.

To identify and characterize the morphophysiological properties of DG-projecting SuM neurons, a retrograde tracer (red retrobeads) was injected into the bilateral DG in the hippocampus (Fig. 1*A*, left) of 3 mice. The injection sites were confirmed by *post hoc* serial coronal sections (Fig. 1*A*, middle). The beads were restricted to the GCL and hilus of the DG (Fig. 1*A*, right top). One week after the injection, the retrogradely labeled DG-projecting neurons were detected primarily in the lateral subdivision of the SuM (SuML) above the mammillothalamic tract (mt) (Fig. 1*A*, right bottom). Only few labeled cells were detected in the medial subdivision of the SuM (SuMM) (Fig. 1*A*, right bottom) as reported previously (Soussi et al., 2010). Notably, in mice injected unilaterally in the right DG (Fig. 1*B*), the labeled DG-projecting SuM cells were mostly detected ipsilateral to the injection side (Fig. 1*C*,*D*; data from 12 slices, 3 mice). Next, we performed whole-cell recordings from labeled DG-projecting SuM neurons located in the SuML in brain slices prepared from both bilateral and unilateral DG-injected mice (Fig. 1*E*). These cells had large cell bodies (≥20 µm in diameter; Fig. 1*E*), with a resting membrane potential of −58.0 ± 1.7 mV (*n* = 11 cells from 5 mice) and an input resistance of 508.3 ± 69.4 mΩ (*n* = 11 cells from 5 mice). They exhibited a bursting firing pattern (at holding potential of −70 mV) in response to small current injection (10-30 pA) and displayed an accommodating firing pattern in response to increased depolarizing current (Fig. 1*F*; *n* = 11 cells from 5 mice). The biocytin-filled SuM cells exhibited axonal projection extending toward the dorsal brain areas with dendrites located within the mammillary region (Fig. 1*G*; *n* = 5 cells from 4 mice).

Next, we used an optogenetic approach to investigate the function of SuM projections. A CaMKIIα-ChR2-eYFP virus was injected into the SuM of WT mice (Fig. 1*H*, top). The SuM neuron projections were observed to form a dense pattern in the supragranular layer of the GCL and CA2 pyramidal layer (Fig. 1*H*, bottom, from 3 mice). To confirm that the ChR2-expressing SuM neurons respond to light stimulation, we made whole-cell recording from these neurons (Fig. 1*I*). When the recorded neurons were illuminated with blue light pulses (470 nm, 5 ms at 5 Hz), they generated spikes in current clamp at −70 mV (Fig. 1*I*, traces; *n* = 7 cells, 5 mice). Similarly, a light-evoked ChR2-mediated inward current was recorded in voltage clamp in the presence of an ionotropic glutamate receptor antagonist, Kyn (2 mm) (Fig. 1*I*, traces). Consistent with previous studies (Boulland et al., 2009; Soussi et al., 2010; Hashimotodani et al., 2018; Root et al., 2018), the ChR2-eYFP-expressing axon terminals in the DG (Fig. 1*J*) coexpressed VGluT2 and VGAT (Fig. 1*K*). A total of 1381 putative boutons (from 9 slices, 2 mice) were identified along the ChR2-eYFP-expressing axons. Overall, 92 ± 1.4% (85%-98%) of the boutons expressed VGluT2, 88 ± 2.3% (82%-97%) expressed VGAT, while 84 ± 2.3% (78%-94%) expressed both VGluT2 and VGAT, similar to previous reports (Soussi et al., 2010; Root et al., 2018; Billwiller et al., 2020).

### SuM input preferentially excites dendrite-targeting INs

**Figure 2.**
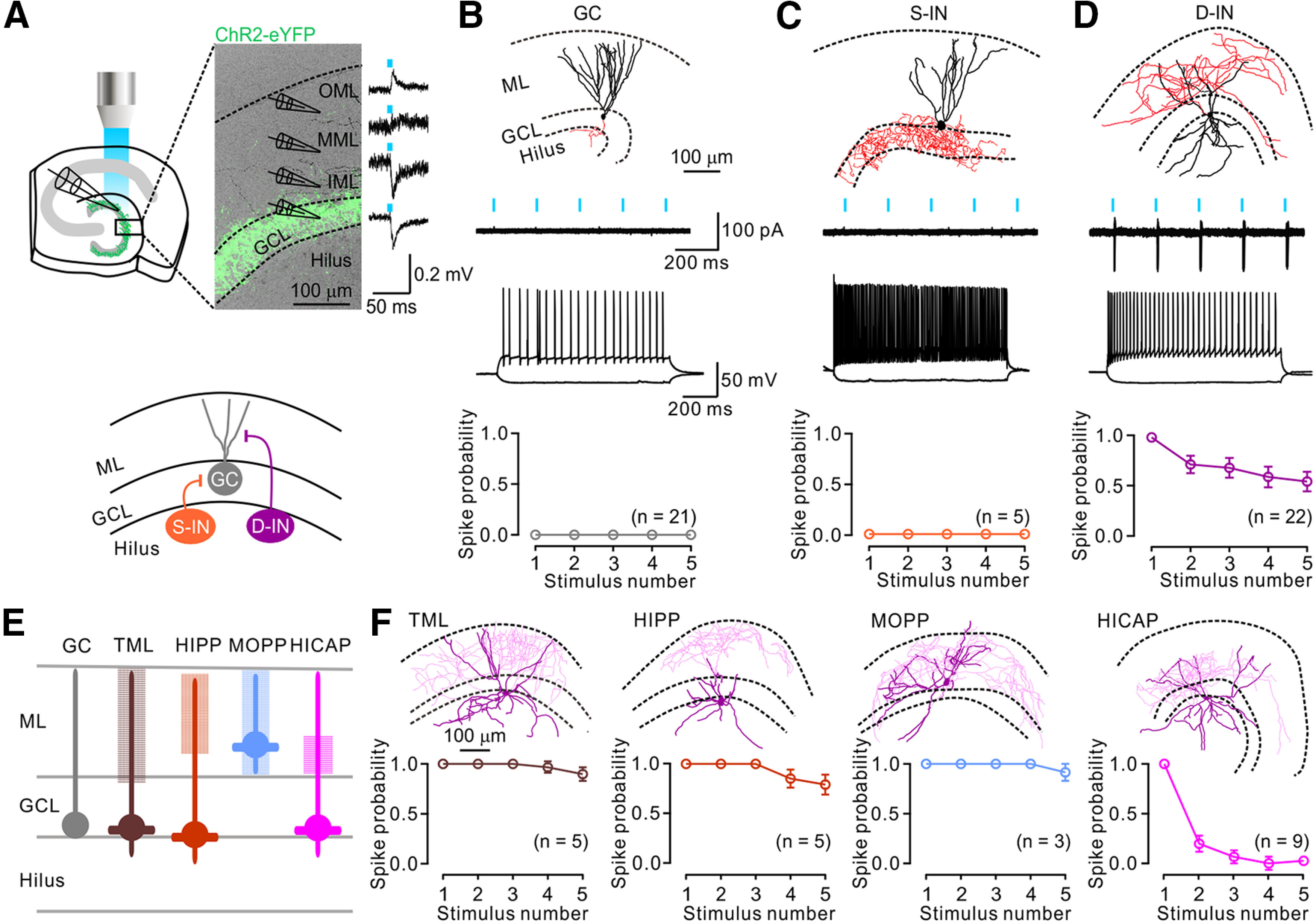
SuM input preferentially excites dendrite-targeting INs in the DG. ***A***, Top, Experimental configuration of LFP recordings and photostimulation. A transverse section across the DG showing ChR2-eYFP-expressing SuM fibers (green) in the GCL and light-evoked LFPs recorded along the somatodendritic axis of GCs in the DG. Bottom, Schematic of local network of the DG depicting GC (gray), S-IN (orange), and D-IN (violet). ***B-D***, Top, Representative morphologic reconstruction of a GC, S-IN, and D-IN (soma and dendrites, black; axon, red) in the DG. Middle, Sample traces of cell-attached responses (six overlaid sweeps) to 5 Hz photostimulation of the SuM input and firing pattern of a representative GC, S-IN, and D-IN. Bottom, Plot of spike probabilities of all recorded cells. ***E***, Summary of identified D-IN subtypes recruited by the SuM input. Filled circles represent soma locations. Thick lines indicate dendrites. Hatched boxes represent axon distribution. ***F***, Top, Morphologic reconstructions of representative TML, HIPP, MOPP, and HICAP in the DG. Bottom, Plot of spike probabilities of recorded cells in response to 5 Hz photostimulation of the SuM input. Error bars indicate mean ± SEM.

Next, we examined SuM-DG synaptic transmission by recording field EPSPs (fEPSPs) along the somatodendritic axis of GCs (Fig. 2*A*, top). The fEPSPs exhibited downward at the GCL (−0.10 ± 0.01 mV; *n* = 7) and inner molecular layer (−0.06 ± 0.01 mV; *n* = 7). The polarity of fEPSP reversed at the middle molecular layer (0.03 ± 0.00 mV; *n* = 7) and exhibited upward at the outer molecular layer (0.03 ± 0.00 mV; *n* = 7). This was consistent with the observation that SuM axons mainly innervated the somatic and proximal dendritic regions of GCs (Hashimotodani et al., 2018). Then, we tested whether activation of SuM terminals alone was sufficient to excite any DG neurons. To this end, we injected a CaMKIIα-ChR2-eYFP virus into the SuM of WT mice or EF1α-DIO-ChR2-eYFP virus into VGluT2-Cre mice. Next, cell-attached recordings were performed from various types of DG neurons, such as GCs, S-INs, and D-INs (Fig. 2*A*, bottom), and followed by biocytin-filled whole-cell recordings for *post hoc* morphologically identification (Liu et al., 2014; Hsu et al., 2016; Lee et al., 2016). Dentate GCs receive coherent theta (4-10 Hz)-band EPSCs *in vivo* (Pernía-Andrade and Jonas, 2014), and the SuM synchronizes with the DG (Li et al., 2020). Thus, we investigated the response of DG cells to SuM activation at a physiologically relevant frequency (e.g., 5 Hz). Upon photostimulation of SuM axons (5 Hz, 5 ms pulses), no spikes were evoked in all recorded GCs (Fig. 2*B*; 21 of 21 cells) and S-INs (Fig. 2*C*; 5 of 5 cells). In contrast, the majority of D-INs reliably generated spikes in response to SuM terminal activation (Fig. 2*D*; 22 of 27 cells). Several morphologic subtypes of D-INs have been well characterized (Freund and Buzsáki, 1996; Hsu et al., 2016). According to their soma locations and the input layers where their axons innervate, there are at least four distinct subtypes, including the total molecular layer cells (TML cells), hilar PP-associated cells (HIPP cells), molecular layer PP-associated cells (MOPP cells), and hilar commissural-associational pathway-related cells (HICAP cells) (Fig. 2*E*). Based on the results of morphologic reconstructions, the spike probability of each subtype was plotted against the stimulus number (Fig. 2*F*). The five nonresponsive D-INs, including two HICAP, two HIPP, and one MOPP, were not included in the plots here. Collectively, the SuM input alone was sufficient to activate most D-INs, but not GCs and S-INs.

### Differential glutamate/GABA cotransmission is target cell-specific

**Figure 3.**
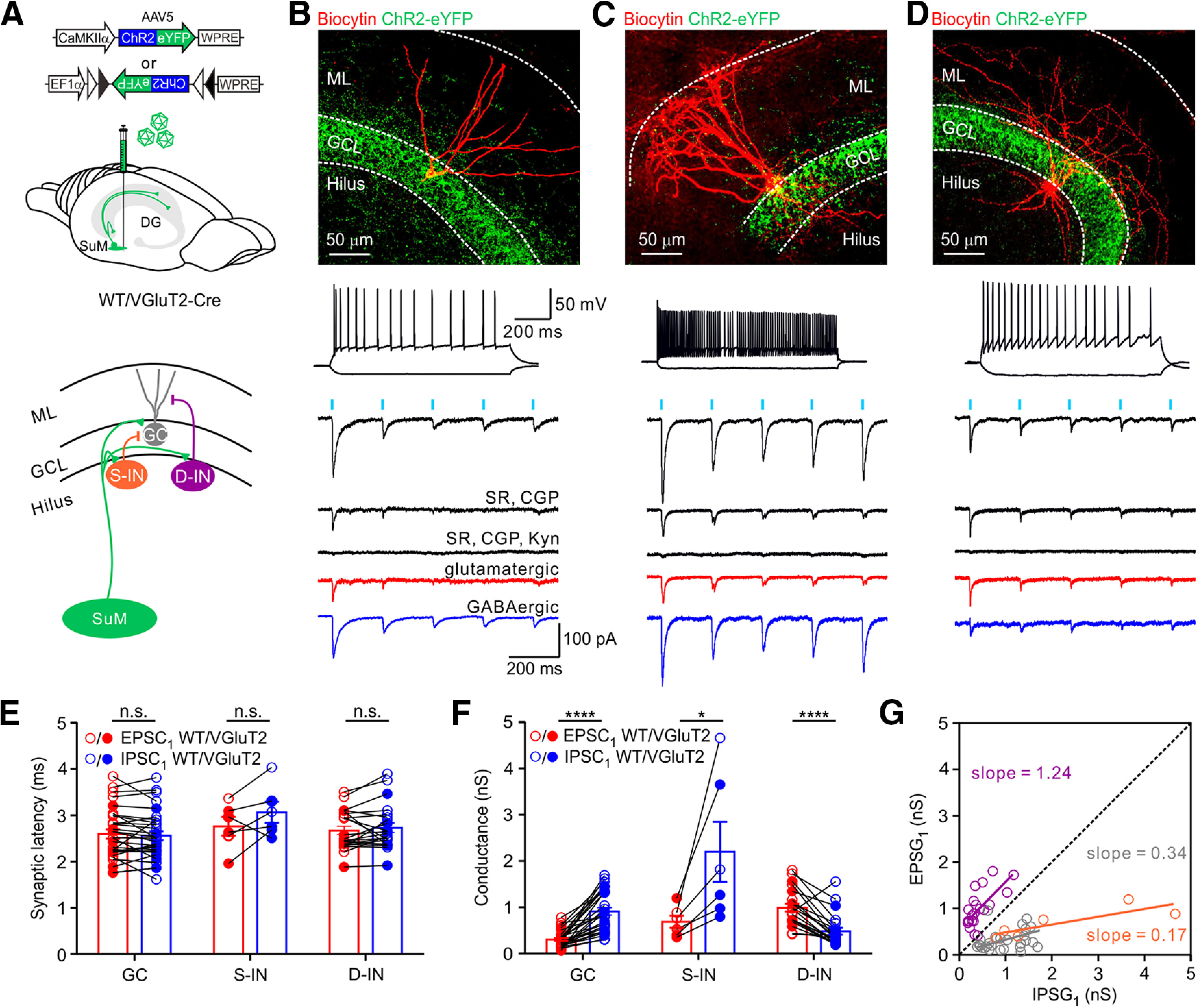
Differential glutamate/GABA cotransmission is target cell-specific. ***A***, Top, Schematic of virus injection into SuM of VGluT2-Cre or WT mouse. Bottom, Schematic of local DG network, including the SuM input (green), GC, S-IN, and D-IN. ***B-D***, Top, Confocal image stacks of transverse sections of the DG depicting selective expression of ChR2-eYFP in the GCL and a biocytin-filled GC, S-IN, and D-IN (red). Middle, Firing pattern of the GC, S-IN, and D-IN. Bottom, Sample traces showing the responses of a GC, S-IN, and D-IN to the 5 Hz photostimulation of the SuM input. Black traces represent average inward currents recorded in ACSF, in the presence of GABA_A_ receptor blocker, SR95531 (1 μm, SR) and GABA_B_ receptor blocker, CGP55845 (1 μm, CGP), and in the presence of SR, CGP, and 2 mm Kyn. The Kyn-sensitive component (glutamatergic, red), and SR & CGP-sensitive component (GABAergic, blue) obtained by digital subtraction from the above traces. ***E***, Plot of synaptic latencies of EPSC_1_ and IPSC_1_ induced by the first light pulse in GCs, S-INs, and D-INs (GCs, EPSC_1_, 2.60 ± 0.10 ms; IPSC_1_, 2.56 ± 0.10 ms; *n* = 30; *p* = 0.875, *U* = 439.0; S-INs, EPSC_1_, 2.78 ± 0.20 ms; IPSC_1_, 3.07 ± 0.23 ms; *n* = 6; *p* = 0.571, *U* = 14.0; D-IN, EPSC_1_, 2.67 ± 0.09; IPSC_1_, 2.73 ± 0.10; *n* = 22; *p* = 0.663, *U* = 223.0; Mann–Whitney test). Circles connected by lines represent data collected from the same cell. Filled circles represent data obtained from VGluT2-Cre line. Open circles represent data from WT mice. ***F***, Plot of excitatory and inhibitory conductances, EPSG_1_ and IPSG_1_ in GCs, S-INs, and D-INs (GCs, EPSG_1_, 0.30 ± 0.03; IPSG_1_, 0.91 ± 0.08; *n* = 30; *p* < 0.0001; *U* = 67.0; S-INs, EPSG_1_, 0.58 ± 0.08 nS; IPSG_1_, 2.14 ± 0.67 nS; *n* = 6; *p* < 0.05; *U* = 4.0; D-INs, EPSG_1_, 0.99 ± 0.08 nS; IPSG_1_, 0.48 ± 0.08 nS; *n* = 22; *p* < 0.0001; *U* = 63; Mann–Whitney test). ***G***, Scatter plot of EPSG_1_ versus IPSG_1_ from GCs (gray circles), S-INs (orange circles), and D-INs (violet circles). Dashed line indicates equality diagonal. Gray, orange, and violet lines indicate the linear regression lines for GCs, S-INs, and D-INs, respectively (slope = 0.34, *R^2^* = 0.20 for GCs; slope = 0.17, *R^2^* = 0.68 for S-INs; and slope = 1.24, *R^2^* = 0.68 for D-INs). Error bars indicate mean ± SEM. *p < 0.05. ****p < 0.0001.

Synaptic excitation and inhibition are critical for neuronal excitability and information processing in neural circuits (Liu, 2004; Yizhar et al., 2011; Bhatia et al., 2019; Iascone et al., 2020). SuM afferents are known to corelease glutamate and GABA onto both GCs and GABAergic INs (Pedersen et al., 2017; Hashimotodani et al., 2018; Li et al., 2020). Given that the SuM input preferentially excites D-INs, we next investigated whether synapse-specific excitation and inhibition correlate with differential recruitment of DG cells. To address this question, ChR2-eYFP was virally expressed in SuM neurons of WT or VGluT2-Cre mice (Fig. 3*A*, top) and recordings were made from transverse slice sections of the DG (Fig. 3*A*, bottom). The expression of ChR2-eYFP in the GCL was confirmed before recordings (Fig. 3*B–D*, top). To determine the synaptic property at the SuM-GC synapse, we performed whole-cell recordings from GCs, which exhibited regular spiking, at −75 mV ([Cl^–^]_i_ = 140 mm; E_GABA_ = ∼0 mV as determined experimentally, Fig. 3*B*, bottom) in brain slices. Photostimulation of the SuM terminals (470 nm, 5 ms at 5 Hz) in the DG-evoked inward currents in all recorded GCs (30 of 30 cells; 12 mice). The mean peak amplitude was 84.0 ± 7.0 pA (*n* = 30) at −75 mV. The mean response was largely reduced by coapplication of a GABA_A_ receptor blocker, SR95531 (1 µm) and a GABA_B_ receptor blocker, CGP55845 (1 µm) to 22.6 ± 2.4 pA and finally almost abolished by Kyn (2 mm) (Fig. 3*B*, traces). The pharmacologically isolated components, SR and CGP-sensitive component (hereafter called “IPSC”) and Kyn-sensitive component (hereafter called “EPSC”), were GABAergic and glutamatergic, respectively (Fig. 3*B*, red trace, EPSC and blue trace, IPSC). The GABAergic component was slower (20%-80% rise time, 2.79 ± 0.37 ms; *n* = 30; decay time constant, 30.67 ± 1.85 ms; *n* = 30) relative to the glutamatergic component (20%-80% rise time, 1.10 ± 0.07 ms; *n* = 30; decay time constant, 6.87 ± 0.47 ms; *n* = 30). Nevertheless, both EPSC and IPSC components exhibited similar synaptic latencies in response to 5 ms photostimulation of SuM terminals (Fig. 3*E*, EPSC_1_, 2.60 ± 0.10 ms; IPSC_1_, 2.56 ± 0.10 ms; *n* = 30; *p* = 0.875, *U* = 439.0; Mann–Whitney test), supporting the idea of glutamate and GABA cotransmission at the SuM-GC synapse. The EPSC and IPSC evoked by SuM terminal activation exhibited strong depression of the amplitude (Fig. 3*B*, bottom traces). Notably, analysis of the first peak excitatory and inhibitory conductances (hereafter called EPSG_1_ and IPSG_1_) revealed that inhibitory transmission dominated at the SuM-GC synapse (Fig. 3*F*, GCs, EPSG_1_, 0.30 ± 0.03 nS; IPSG_1_, 0.91 ± 0.08 nS; *n* = 30; *p* < 0.0001; *U* = 67.0; Mann–Whitney test). Moreover, the scatter plot of individual relationship between EPSG_1_ and IPSG_1_ obtained from each cell showed a bias toward IPSG (Fig. 3*G*, gray circles), and the slope of the linear regression line (gray line) was <1. Together, GABAergic transmission was predominant at the SuM to GC synapse.

Next, we investigated the synaptic property of different IN subtypes (Fig. 3*C*,*D*). Photostimulation of the SuM terminals evoked variable inward currents (Fig. 3*C*,*D*, black traces) in different IN subtypes. Similar to GCs, the evoked postsynaptic current recorded from putative S-INs, which exhibited fast-spiking firing pattern (see Materials and Methods). In our recording, S-INs exhibited a maximum firing rate of 74.0 ± 4.9 Hz (*n* = 6 cells; 5 mice), largely blocked by bath application of SR95531 (1 µm) and CGP55845 (1 µm) (Fig. 3*C*, bottom traces). The remaining small excitatory component was blocked by Kyn (2 mm). Overall, 3 of 6 fast-spiking INs were morphologically confirmed as S-INs. The pharmacologically isolated EPSCs and IPSCs in S-INs have similar synaptic latencies (Fig. 3*E*, S-INs, EPSC_1_, 2.78 ± 0.20 ms; IPSC_1_, 3.07 ± 0.23 ms; *n* = 6; *p* = 0.571, *U* = 14.0; Mann–Whitney test). The 20%-80% rise time of the IPSC and EPSC was 1.73 ± 0.31 ms and 1.19 ± 0.08 ms (*n* = 6), respectively, whereas the decay time constant of IPSC and EPSC was 17.40 ± 1.53 ms and 7.94 ± 0.55 ms (*n* = 6), respectively. Like SuM-GC synapses, analysis of EPSG_1_ and IPSG_1_ showed that inhibitory conductance dominated at the SuM-S-IN synapses (Fig. 3*F*, S-INs, EPSG_1_, 0.58 ± 0.08 nS; IPSG_1_, 2.14 ± 0.67 nS; *n* = 6; *p* < 0.05; *U* = 4.0; Mann–Whitney test). However, the IPSGs at SuM-S-IN synapses were larger than that at SuM-GC synapses (S-INs, IPSG_1_, 2.14 ± 0.67 nS; GCs, IPSG_1_, 0.91 ± 0.08 nS, *p* < 0.01, unpaired *t* test). Furthermore, the plot of EPSG_1_ versus IPSG_1_ showed a bias toward the IPSG_1_, confirming the dominance of inhibitory conductance at the SuM-S-IN synapses (Fig. 3*G*, orange regression line).

**Figure 4.**
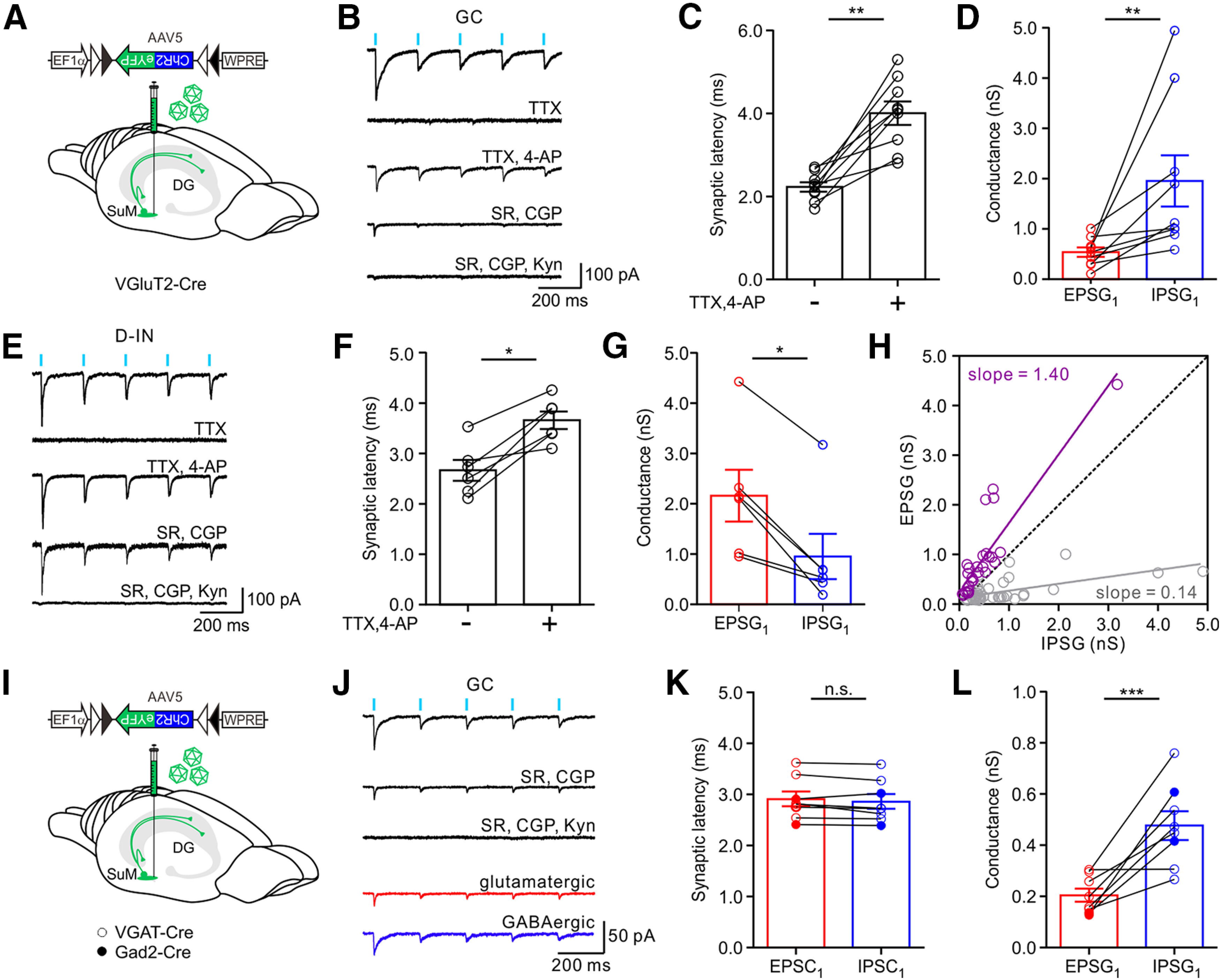
SuM input forms monosynaptic connections with GCs and D-INs. ***A***, Schematic of virus injection into the SuM of VGluT2-Cre mice. ***B***, Representative traces of light-evoked responses recorded from a GC in ACSF, TTX (1 μm), and TTX, 4-AP (1 mm). TTX completely block the response and recovered by 4-AP. Addition of SR (1 μm) and CGP (1 μm) largely blocked the response; Kyn (2 mm) completely abolished the remaining responses. ***C***, Synaptic latencies before and after bath application of TTX, 4-AP at the SuM-GC synapse; ACSF, 2.24 ± 0.11 ms; TTX, 4-AP, 4.01 ± 0.28 ms; *n* = 9; *p* = 0.0039, Wilcoxon sign-rank test. ***D***, Plot of EPSG_1_ and IPSG_1_ of GCs. EPSG_1_, 0.53 ± 0.10 nS; IPSG_1_, 1.95 ± 0.51 nS; *n* = 9; *p* = 0.0012; *U* = 6.0; Mann–Whitney test. ***E***, Representative traces of light-evoked responses recorded from a D-IN in ACSF, TTX (1 μm), and TTX (1 μm) & 4-AP (1 mm). TTX completely block the response and recovered by 4-AP. SR (1 μm) and CGP (1 μm) slightly block the response, and finally, Kyn (2 mm) completely abolished the remaining responses. ***F***, Plot of synaptic latencies before and after bath application of TTX, 4-AP at the SuM-D-IN synapses; ACSF, 2.67 ± 0.21 ms; TTX & 4-AP, 3.66 ± 0.17 ms; *n* = 6, *p* = 0.0313, Wilcoxon sign-rank test. ***G***, Plot of EPSG_1_ and IPSG_1_ of D-INs. EPSG_1_, 2.16 ± 0.51 nS; IPSG_1_, 0.95 ± 0.06 nS; *n* = 6; *p* = 0.0411; *U* = 5.0; Mann–Whitney test. ***H***, Scatter plot of EPSG versus IPSG from GCs (gray circles) and D-INs (violet circles) during 5 Hz photostimulation of SuM input. Dashed line indicates equality diagonal. Gray and violet lines indicate the linear regression lines for GCs and D-INs, respectively (slope = 0.14, *R^2^* = 0.40 for GCs; and slope = 1.40, *R^2^* = 0.78 for D-INs). Error bars indicate mean ± SEM. ***I***, Schematic of virus injection into the SuM of VGAT-Cre (open circle) and Gad2-Cre (closed circle) mice. ***J***, Sample traces showing the responses of a GC to the 5 Hz photostimulation of the SuM input. Black trace represents average inward currents recorded in ACSF, in the presence of GABA_A_ receptor blocker, SR95531 (1 μm, SR) and GABA_B_ receptor blocker, CGP55845 (1 μm, CGP), and in the presence of SR, CGP, and 2 mm Kyn. The Kyn-sensitive component (glutamatergic, red), and SR & CGP-sensitive component (GABAergic, blue) obtained by digital subtraction from the above traces. ***K***, Plot of synaptic latencies of EPSC_1_ and IPSC_1_ of GCs. EPSC_1_, 2.91 ± 0.14 nS; IPSC_1_, 2.86 ± 0.14 nS; *n* = 8; *p* = 0.5604; *U* = 26.0; Mann–Whitney test. ***L***, Plot of conductances EPSG_1_ and IPSG_1_ of GCs. EPSG_1_, 0.20 ± 0.03 nS; IPSG_1_, 0.48 ± 0.06 nS; *n* = 8; *p* = 0.0006; *U* = 2.0; Mann–Whitney test. *p < 0.05. **p < 0.01. ***p < 0.001.

**Figure 5.**
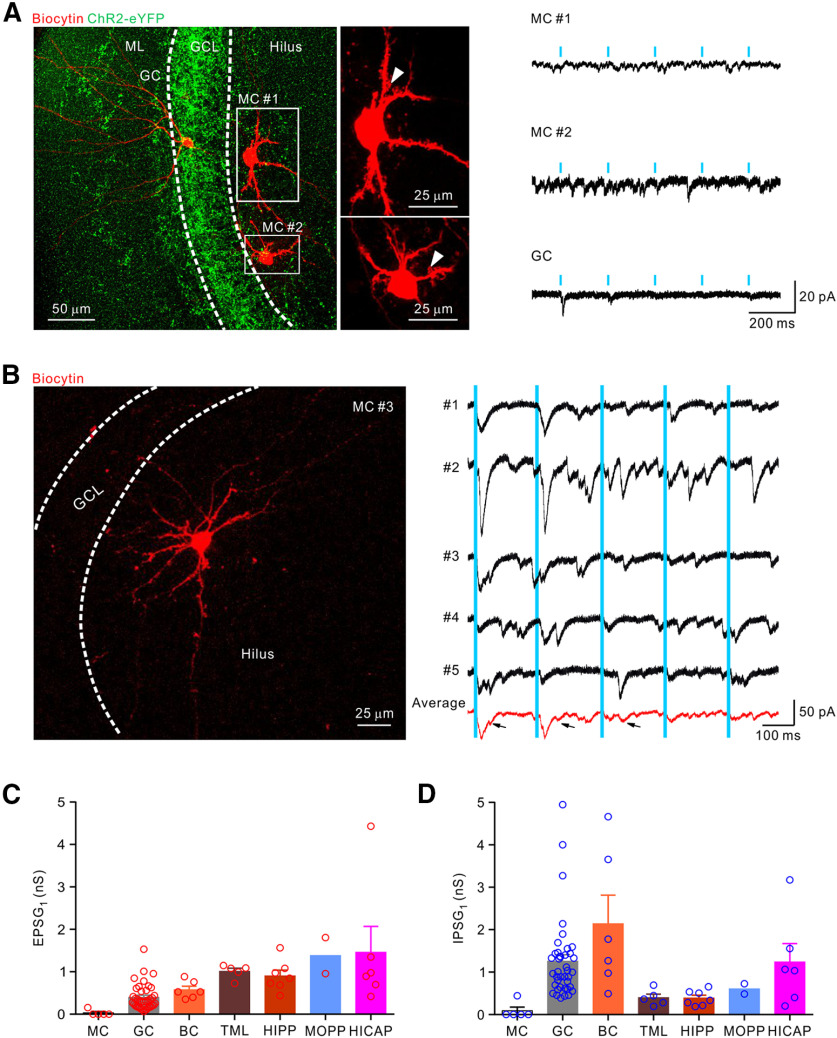
MCs receive weak synaptic input from the SuM. ***A***, Left, Confocal image stacks of transverse sections through the DG depicting selective expression of ChR2-eYFP in VGluT2^+^ SuM fibers (green) in the GCL and sequentially recorded biocytin-filled MC #1, MC #2 (arrowheads, thorny excrescences), and a GC. Right, Representative traces obtained from MC #1, MC #2, and a GC in response to the photostimulation of the SuM input. ***B***, Left, Morphology of a biocytin-filled responsive MC #3. Right, Black traces represent individual traces of responses of the MC #3 to 5 Hz photostimulation of SuM input. Red trace represents the average trace. Arrows indicate disynaptic responses. ***C***, ***D***, Summary of the EPSG_1_ and IPSG_1_, respectively, recorded from different cell types in the DG. Circles represent individual cells. Error bars indicate mean ± SEM.

Intriguingly, unlike GCs and S-INs, the coapplication of GABA_A_ and GABA_B_ receptor blockers SR95531 (1 µm) and CGP55845 (1 µm) slightly reduced the postsynaptic current recorded in most D-INs (Fig. 3*D*, bottom). However, further bath application of Kyn completely blocked the remaining large current, indicating a dominant excitatory transmission at the SuM-D-IN synapses (Fig. 3*D*). The pharmacologically isolated EPSC and IPSC (Fig. 2*D*; EPSC, red trace and IPSC, blue trace) exhibited similar synaptic latencies (Fig. 3*E*, D-INs, EPSC_1_, 2.67 ± 0.09 ms; IPSC_1_, 2.73 ± 0.10 ms; *n* = 22; *p* = 0.663, *U* = 223.0; Mann–Whitney test). The IPSC kinetics was slower (20%-80% rise time, 2.53 ± 0.23 ms; *n* = 25; decay time constant, 19.64 ± 2.40 ms; *n* = 25) relative to the EPSC kinetics (20%-80% rise time, 1.25 ± 0.11 ms; *n* = 25; decay time constant, 6.38 ± 0.61 ms; *n* = 25). Contrary to the SuM-GC and SuM-S-IN synapses, analysis of EPSG_1_ and IPSG_1_ showed that excitation dominated the SuM-D-IN synapses (Fig. 3*F*, D-INs, EPSG_1_, 0.99 ± 0.08 nS; IPSG_1_, 0.48 ± 0.08 nS; *n* = 22; *p* < 0.0001; *U* = 63; Mann–Whitney test). The plot of EPSG versus IPSG recorded from each cell revealed a clear shift toward excitatory conductance (Fig. 3*G*, violet circles), and the slope was >1 (Fig. 3*G*). In another set of experiments of VGluT2-Cre mouse virally injected with EF1α-DIO-ChR2-eYFP (Fig. 4*A*), the monosynaptic cotransmission of the glutamate and GABA was also pharmacologically verified by adding TTX, a voltage-dependent sodium channel blocker, and 4-aminopyridine (4-AP), a voltage-dependent potassium channel blocker (Fig. 4*B*, GC and Fig. 4*E*, D-IN). The light-evoked postsynaptic current was completely abolished by bath application of TTX (1 µm) and was reversed by subsequent addition of 4-AP (1 mm; in the presence of TTX). Consistent with a previous report (Hsu et al., 2016), synaptic latencies were significantly increased by 4-AP (Fig. 4*C*; SuM-GC; synaptic latency, baseline, 2.24 ± 0.11 ms ms; TTX & 4-AP, 4.01 ± 0.28 ms; *n* = 9 cells; 5 mice; Fig. 4*F*, SuM-D-IN; synaptic latency, baseline, 2.67 ± 0.21 ms; TTX & 4-AP, 3.66 ± 0.17 ms; *n* = 6 cells; 4 mice). Analysis of the EPSG and IPSG further confirmed that GABAergic transmission dominated at the SuM-GC synapse (Fig. 4*D*), whereas glutamatergic transmission was predominant at the SuM-D-IN synapse (Fig. 4*G*). Moreover, the scatter plot of all EPSGs and IPSGs obtained from individual cells revealed a slop of 0.14 at the SuM-GC synapse and a slop of 1.40 at the SuM-D-IN synapses (Fig. 4*H*). Similar results were obtained from GCs recorded in VGAT-Cre and Gad2-Cre transgenic mice (Fig. 4*I–L*). In addition to GCs and INs, we also checked the functional connectivity between the SuM input and mossy cells (MCs), which are excitatory neurons located in the hilus and featured by prominent thorny excrescences at their proximal dendrites (Fig. 5*A*). We performed sequential whole-cell recordings from GCs and MCs (Fig. 5*A*). Consistent with a recent report that MCs rarely receive synaptic input from SuM (Hashimotodani et al., 2018), only 1 of 5 MCs (4 mice) recorded received the discernible response to photostimulation of the SuM input (Fig. 5*D*), and the current was small (−42 pA; Fig. 5*D*). The summary plots of first EPSG and IPSG obtained from different cell types in the DG are shown in Figure 5*C*, *D*.

**Figure 6.**
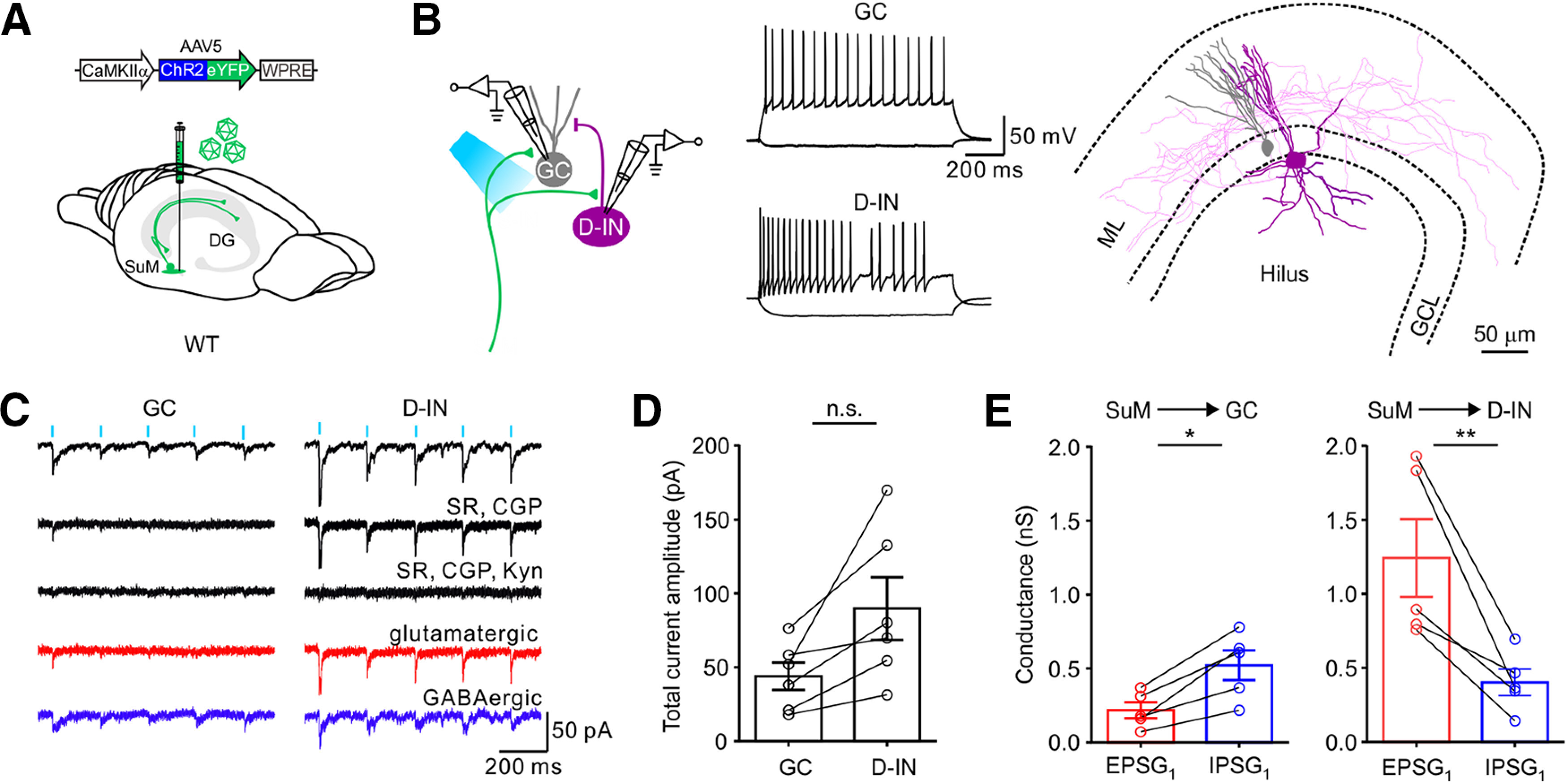
Synaptic responses from simultaneously recorded GCs and D-INs. ***A***, Schematic of virus injection into the SuM. ***B***, Left, Simultaneous whole-cell recording from a GC and a D-IN. Middle, The firing pattern of the recorded GC and D-IN. Right, The morphologic reconstruction of GC (gray) and D-IN (violet). ***C***, Traces of light-evoked postsynaptic responses recorded in GC and D-IN in baseline, SR & CGP, SR, GCP & Kyn, glutamatergic component (red), and GABAergic component (blue). ***D***, Plot of the total composite current amplitude in the GCs and D-INs simultaneously recorded. GC, 43.94 ± 9.24 pA; D-IN, 89.78 ± 21.13 pA; *n* = 6; *p* = 0.0931; *U* = 7.0; Mann–Whitney test. Circles connected by dashed lines represent data collected from cells recorded simultaneously from the same slice. ***E***, Plot of conductances of EPSG and IPSG at the SuM-GC and SuM-D-IN synapses. SuM-GC, EPSG_1_, 0.22 ± 0.05 nS; IPSG_1_, 0.52 ± 0.10 nS; *n* = 5; *p* < 0.05; *U* = 2.0; SuM-D-IN, EPSG_1_, 1.24 ± 0.26 nS; IPSG_1_, 0.40 ± 0.09 nS; *n* = 5; *p* < 0.01; *U* = 0.0; Mann–Whitney test. *p < 0.05. **p < 0.01.

Finally, to exclude the possibility that the distinct synaptic properties observed here were because of variable viral expression from slices to slices, we performed another set of experiments in WT mice (Fig. 6*A*), where simultaneous dual recordings of GCs and D-INs were obtained from the same slices (Fig. 6*B*). We found that photostimulation of SuM input (5 ms, 470 nm, 5 Hz light pulses) in the DG evoked inward currents in both GCs and INs (Fig. 6*C*, black traces, 6 of 7 pairs recorded). Coapplication of SR95531 (1 µm) and CGP55845 (1 µm) blocked ∼70.5 ± 5.0% of current in GCs, only ∼25.5 ± 5.5% was blocked in D-INs, and Kyn (2 mm) completely blocked the remaining current in both GCs and D-INs (Fig. 6*C*). The synaptic strength was stronger at the SuM-D-INs synapses compared with that at the SuM-GC synapses (Fig. 6*D*). Consistent with this, analysis of the peak excitatory and inhibitory conductances (EPSG_1_ and IPSG_1_) in some cells revealed that inhibitory transmission dominated at the SuM-GC synapses (Fig. 6*E*, left, EPSG_1_; 0.22 ± 0.05 nS, IPSG_1_; 0.52 ± 0.10 nS; *n* = 5 cells; 4 mice; *p* < 0.05; *U* = 2.0; Mann–Whitney test), while excitatory transmission dominated at the SuM-D-IN synapses (Fig. 6*E*, right, EPSG_1_; 1.24 ± 0.26 nS, IPSG_1_; 0.40 ± 0.09 nS; *n* = 5 cells; 4 mice; *p* < 0.01; *U* = 0.0; Mann–Whitney test). Together, these results demonstrated that the ratio of excitatory and inhibitory components at SuM-DG synapses depends on the subtypes of target cells.

### SuM input shortens spike latency and enhances spike-timing precision

**Figure 7.**
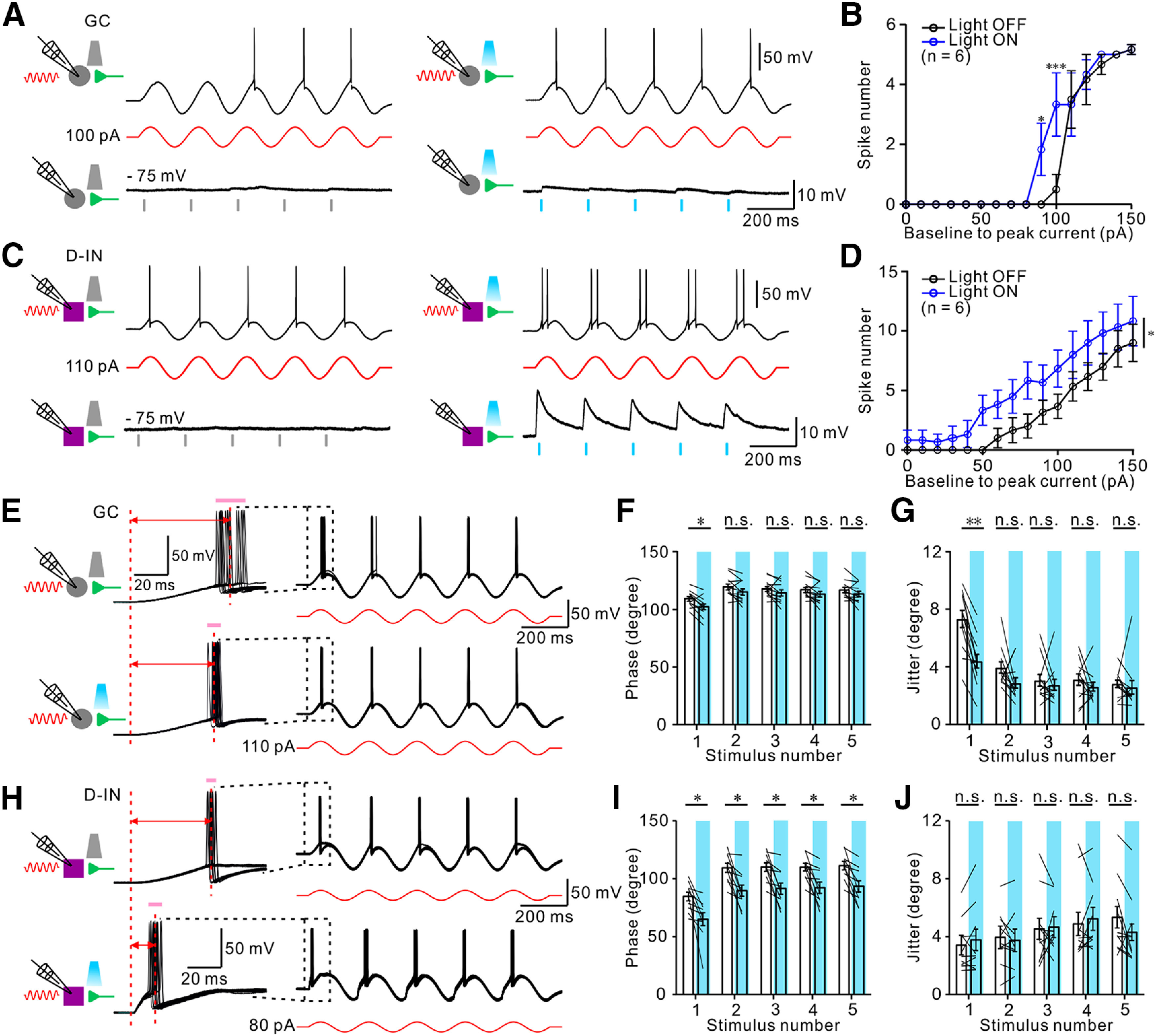
SuM input shortens spike latency and enhances spike-timing precision. ***A***, Top, Representative traces of responses of GCs to sinusoidal current steps before (left) and after (right) photostimulation of SuM input. Middle, Baseline to peak current amplitude of 100 pA sinusoidal protocol (red traces). Bottom, EPSP evoked by photostimulation of SuM input. Gray bars represent light off. Blue bars represent time of photostimulation at 5 Hz. ***B***, Plot of spike number versus baseline to peak current in GCs. ***C***, Top, Representative traces of responses of D-INs to sinusoidal current steps before (left) and after (right) photostimulation of SuM input. Middle, Baseline to peak current amplitude of 110 pA sinusoidal protocol (red traces). Bottom, EPSP evoked by photostimulation of SuM input. Blue bars represent time of photostimulation at 5 Hz. ***D***, Plot of spike number versus baseline to peak current in D-INs. ***E***, Representative traces of responses of GCs (20 overlaid sweeps) to constant suprathreshold sinusoidal current injection without (top traces) and with (bottom traces) photostimulation of SuM input. Left, Enlarged traces of action potentials induced by first stimulus without (top traces) and with photostimulation of SuM input (bottom traces). Red dotted lines and the red arrow lines indicate a shift in the mean spike latencies between onset of sinusoid current injection and the mean time point of peak in each action potential. Pink bars represent spike jitters. ***F***, Summary plot of spike phase. *n* = 12; *F*_(4,44)_ = 20.43; *p* < 0.0001; two-way ANOVA with Bonferroni *post hoc* test. Error bars indicate mean ± SEM. ***G***, Summary plot of spike jitter. *n* = 12; *F*_(4,44)_ = 22.17; *p* < 0.0001; two-way ANOVA with Bonferroni *post hoc* test. ***H***, Representative traces of responses of D-INs (20 overlaid sweeps) to constant suprathreshold sinusoidal current injection without (top traces) and with (bottom traces) photostimulation of the SuM input. Left, Enlarged traces of action potentials induced by first stimulus without (top traces) and with photostimulation (bottom traces). Red dotted lines and the red arrow lines indicate a shift in the mean spike latencies between onset of sinusoid current injection and the mean time point of peak in each action potential. Pink bars represent spike jitters. ***I***, Summary plot of spike phase. *n* = 10; *F*_(4,36)_ = 115.4; *p* < 0.0001; two-way ANOVA with Bonferroni *post hoc* test. Error bars indicate mean ± SEM. ***J***, Summary plot of spike jitter. *n* = 10; *F*_(4,36)_ = 5.0; *p* = 0.0027; two-way ANOVA with Bonferroni *post hoc* test. *p < 0.05. **p < 0.01.

Cortical principal neurons fire with large variability in response to identical stimuli *in vivo* (Shadlen and Newsome, 1998; Fricker and Miles, 2001; Carandini, 2004). Well-timed inhibition from GABAergic transmission is known to promote precise spike timing, which is essential for hippocampal network oscillation and is thought to be critical for several cognitive functions (Bacci and Huguenard, 2006; Woodruff and Sah, 2007; Hou et al., 2016). Here, we explored how SuM-driven synaptic excitatory and inhibitory conductances regulate spike generation in GCs and D-INs using the low chloride internal solution [Cl^–^]_i_ = 7.2 mm, which is close to the physiological intracellular chloride concentration (Chiang et al., 2012). To simulate *in vivo* membrane oscillations, GCs and D-INs were driven by injecting sinusoidal current steps at low theta (5 Hz) frequencies (Fig. 7). Under this condition, photostimulation of the SuM input at the ascending phase of each theta cycle slightly increased spike numbers in GCs (Fig. 7*A*,*B*; see Materials and Methods). Given that D-INs received predominantly synaptic excitation on SuM activation, we next examined the modulatory effect of SuM activation on spike generation in D-INs in response to the same oscillatory input. Compared with the light-off epoch, photostimulation of the SuM input remarkably increased spike numbers in D-INs in response to sinusoidal current injections (Fig. 7*C*,*D*). Next, we examined the latency and spike jitter in GCs and D-INs by injecting a constant suprathreshold sinusoidal current, which was near enough to generate single spikes near the peak of each theta cycle (GCs, Fig. 7*E*; D-INs, Fig. 7*H*). Superimposition of spike trains from GCs (Fig. 7*E*) showed that SuM stimulation shortened the spike latencies and decreased spike jitters (Fig. 7*E*, traces). Both reduction in spike latencies and jitters were only significant in first spike (Fig. 7*F*,*G*), which could be explained by strong synaptic depression at the SuM to GC synapses. Notably, superimposition of spike trains from D-INs showed that pairing the SuM input with the suprathreshold sinusoidal stimulation (baseline-to-peak current amplitude of 80 pA) greatly reduced spike latencies (Fig. 7*I*). In great contrast to GCs, photostimulation of the SuM input did not have a significant effect on spike jitters in D-INs (Fig. 7*J*). This result was consistent with our observation of high synaptic excitation and low synaptic inhibition at the SuM-D-IN synapses. Together, activation of SuM input differentially regulates spike generation in GCs and D-INs.

### SuM input enhances GC excitability, thereby supporting LTP

**Figure 8.**
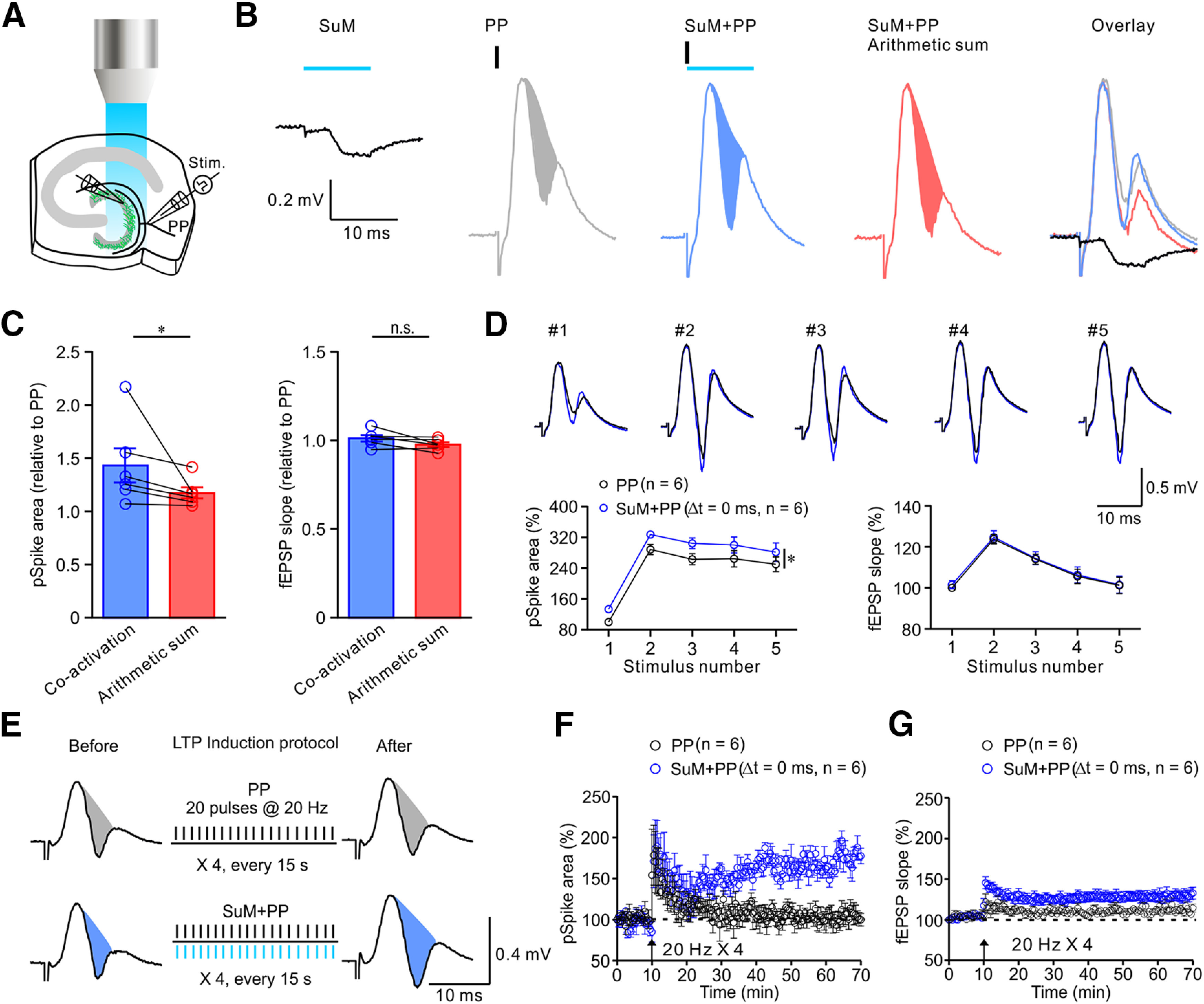
SuM input promotes GC responses to cortical input, thereby enhancing LTP at the PP-GC synapses. ***A***, Experimental schematic showing a stimulation electrode (stim.) placed in the subiculum to electrically activate the PP fibers, a field-recording electrode in the GCL to monitor LFP and pSpike, and a blue light for photostimulation of the SuM axon terminals in the GCL. ***B***, Representative traces of SuM-mediated fEPSP (black trace) after photostimulation, PP-mediated pSpike (filled area in gray) on electrical stimulation, and a pSpike (filled area in light blue) after the coactivation (Δ*t* = 0 ms) of the SuM and PP. Red represents the arithmetic sum of fEPSP and pSpike. The traces of pSpikes were superimposed and aligned with fEPSP. ***C***, Left bar graph, Summary plots of the pSpike areas evoked by SuM+PP coactivation (light blue) and arithmetic sum of SuM-evoked fEPSP and PP-evoked pSpike (light red). Areas were normalized to pSpike area evoked by the PP alone. SuM+PP coactivation, 1.43 ± 0.16; SuM+PP arithmetic sum, 1.17 ± 0.05; *n* = 6; *p* = 0.0313. Right bar graph, Summary plots of relative fEPSP slope, SuM+PP coactivation, 1.01 ± 0.02; SuM+PP arithmetic sum, 0.97 ± 0.01; *n* = 6; Wilcoxon signed-rank test. ***D***, Top, Representative traces of pSpike responses to PP stimulation alone (black traces) and SuM+PP (blue traces) during a 5 Hz train. Bottom left, Summary of the effect of SuM activation on PP-evoked pSpikes versus stimulus number. PP, *n* = 6; PP + SuM, *n* = 6; *p* < 0.05; two-way ANOVA with Bonferroni *post hoc* test. Right, fEPSP slope before and after photostimulation of the SuM input. PP, *n* = 6; SuM+PP, *n* = 6; two-way ANOVA with Bonferroni *post hoc* test. ***E***, Left, Representative traces of baseline pSpikes in response to stimulation of PP alone. Middle, LTP induction protocol consisting of four trains of 20 Hz electrical stimulation of the PP alone at 15 s intertrain interval (top) or coactivation of the PP and 20 Hz, 4 trains, 10 ms photostimulation of the SuM input (bottom). Right, Sample traces of pSpikes after LTP induction. ***F***, Time course of the normalized pSpike area recorded from the GCL in response to 20 Hz, 4 trains stimulation of PP inputs alone (black circles), or coactivation of the PP input stimulation and 20 Hz photostimulation of the SuM input (blue circles). PP alone, 104.8 ± 8.59%; *n* = 6; SuM+PP, 167.6 ± 5.30%; *n* = 6; *p* = 0.0009; paired *t* test. ***G***, Time course of the normalized fEPSP slope of pSpikes recorded from the GCL in response to 20 Hz, 4 train stimulation of PP inputs alone (black circles), or coactivation of PP input stimulation with 20 Hz photostimulation of the SuM input (blue circles). PP alone, 110.6 ± 2.20%; *n* = 6; SuM+PP, 128.5 ± 5.19%; *n* = 6; *p* = 0.0598; paired *t* test. Error bars indicate mean ± SEM. *p < 0.05.

Subcortical inputs modulate GC responses to cortical inputs *in vivo* (Nakanishi et al., 2001; Li et al., 2020). In the DG circuits, the equilibrium potential of GABAergic conductance (E_GABA_) is ∼−72 mV (Chiang et al., 2012), which is more depolarized than the resting potential of GCs (ranging from −80 to −90 mV). Thus, GABA, which is cotransmitted with glutamate by the SuM, could exert either the “shunting inhibitory” or “depolarizing (or excitatory)” effect on GCs. Our previous studies (Chiang et al., 2012; Hsu et al., 2016) report that GABA could promote action potential generation in GCs. Next, we investigated the functional relevance of glutamate/GABA cotransmission on GC responses to the excitatory PP input. We performed LFP recordings in the GCL in response to photostimulation of the SuM input and/or electrical stimulation of the PP input (Fig. 8*A*). The evoked response consisted of the fEPSP and pSpike, a proxy of synaptic strength and GC activity, respectively. Photostimulation of the SuM input evoked the fEPSP but did not generate the pSpike (Fig. 8*B*, black trace), whereas electrical stimulation of the PP generated a compound response, which consisted of the fEPSP followed by the pSpike (Fig. 8*B*, gray area trace). Notably, paired activation of the PP and SuM inputs significantly increased the pSpike area (Fig. 8*B*, blue area trace), indicating an increase in GC spike numbers. The summated trace obtained by digital summation of SuM-fEPSP and PP response was shown in the red trace (Fig. 8*B*, arithmetic sum). Finally, we overlaid all traces and revealed that the SuM-fEPSP emerged before the onset of pSpikes (Fig. 8*B*, overlay). In sum, the pSpike area induced by coactivation of SuM and PP inputs was significantly larger than that of summated trace (Fig. 8*C*, left). Notably, there was no significant change in the relative slope of fEPSP (Fig. 8*C*, right). Further analysis of successive GC responses to either PP activation alone or coactivation of PP and SuM during the 5 Hz trains (Fig. 8*D*, top traces) showed significant increases in the pSpike area (Fig. 8*D*, bottom left plot), but not in the fEPSP slope (Fig. 8*D*, bottom right plot). The lack of changes in the fEPSP slope during coactivation of PP and SuM supports the anatomic finding that SuM axons preferentially innervate the proximal part of GC dendrites.

We hypothesize that the excitatory effect of SuM activation on GCs could enhance LTP induction. To test this hypothesis, we stimulated the cortical input to GCs using a weak protocol (e.g., 20 Hz train stimulation) without and with SuM activation (Fig. 8*E*). After train stimulation, we measured the changes in the synaptic responses. For the SuM + PP protocol, the electrical stimulation of the PP and photostimulation of the SuM input were timed to occur simultaneously (Δ*t* = 0 ms; Fig. 8*E*, left). The pSpikes were monitored after induction of LTP (Fig. 8*E*). Notably, 20 Hz PP stimulation alone could not induce LTP (black circles); however, pairing it with photostimulation of the SuM input (20 Hz, 4 trains, 470 nm, 10 ms) resulted in an increase in pSpike and fEPSP slope (Fig. 8*F*,*G*). Collectively, the SuM input enhanced GC responses to cortical inputs, thereby facilitating induction of LTP at the PP-GC synapses.

## Discussion

Glutamate and GABA are packed in distinct vesicles at the SuM terminals (Boulland et al., 2009; Root et al., 2018). Therefore, the loading, release, and recycling of these two neurotransmitters at the SuM terminals are likely to be regulated differentially. In this study, we demonstrated that glutamate/GABA coreleasing SuM neurons establish synapses with GCs and various subtypes of GABAergic INs in the DG. Notably, the synaptic excitation and inhibition at the SuM-DG synapses are target-specific. SuM-GC and SuM-S-IN synapses are predominantly GABAergic, whereas SuM-D-IN synapses are mainly glutamatergic in nature.

The target cell-dependent excitation and inhibition at the SuM-DG synapses may be important for precise processing of neural information (Liu, 2004; Turrigiano and Nelson, 2004). We demonstrated a dominant inhibitory transmission at the SuM-S-IN synapses (Fig. 3*C*), which might be responsible for weak disynaptic somatic inhibition in GCs (Hashimotodani et al., 2018). Feedforward inhibition is believed to enhance spike timing precision by curtailing EPSPs (Pouille and Scanziani, 2001). The reduced disynaptic feedforward inhibition appears to be compensated by cotransmission of GABA along with glutamate at SuM-GC synapses. The imbalance of synaptic excitation and inhibition has been associated with neurologic disorders, including epilepsy, autism spectrum disorders, schizophrenia, addiction, depression, and social dysfunction (Yizhar et al., 2011; Shabel et al., 2014; Meye et al., 2016). Consistent with this notion, the SuM fibers in the supragranular layer extend aberrant axonal sprouting to the inner molecular layer and are mostly VGluT2^+^ in an epileptic rat model (Soussi et al., 2015).

### A proposed modulatory role of SuM in the DG network

**Figure 9.**
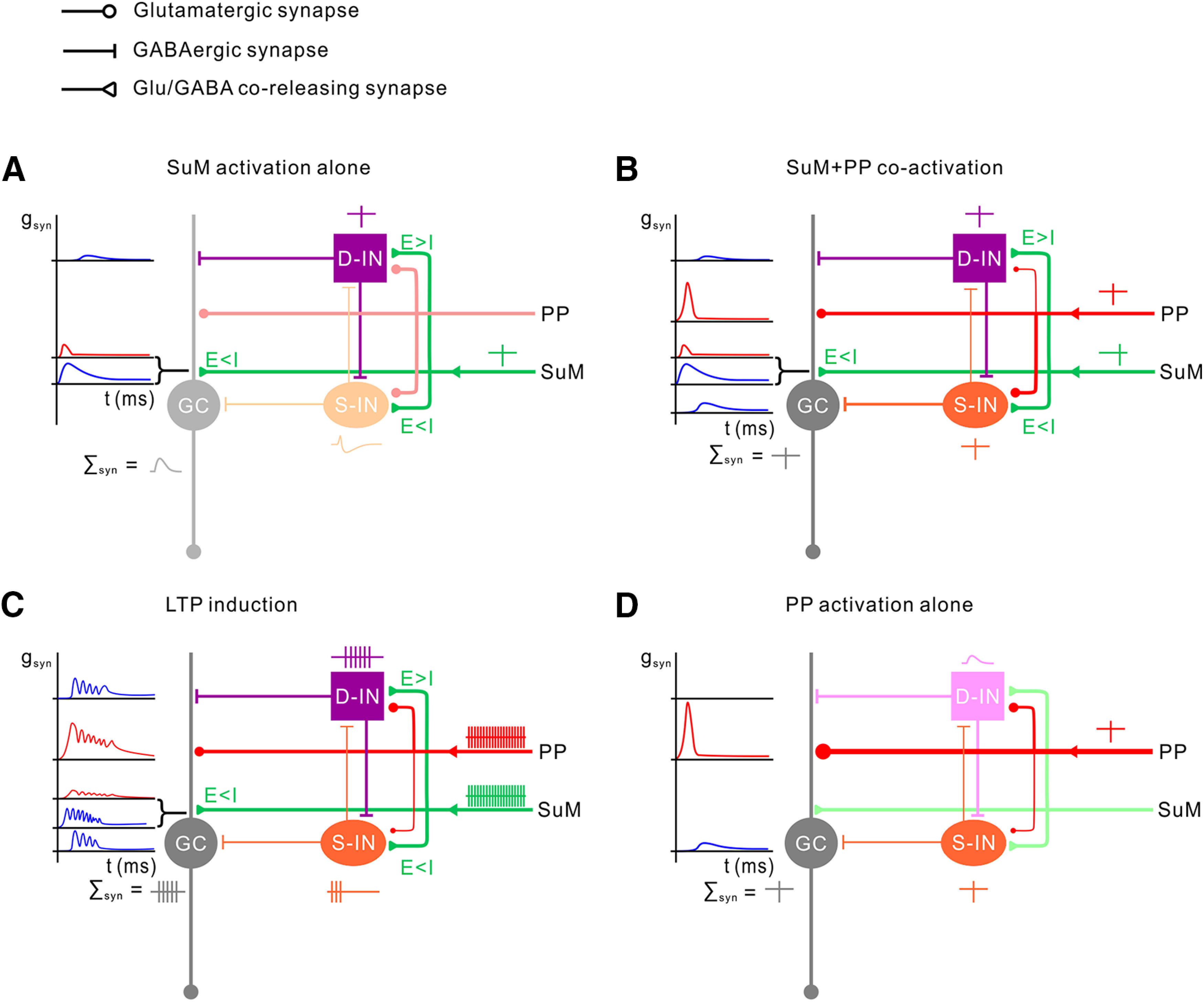
A proposed modulatory role of SuM input in the DG network. ***A***, Schematic of the DG network model showing the synapses between the SuM input (green) and the GC (gray), the D-IN (violet square), and S-IN (orange oval). The SuM input forms monosynaptic excitatory and inhibitory connections with the GC, D-IN, and S-IN. At SuM-GC and SuM-S-IN, E < I; while at SuM-D-IN, E > I. Activation of SuM input (green action potential) results in spike generation in D-IN (violet action potential), but only subthreshold depolarization in the GCs and S-INs. The synaptic summation in this model leads to a small subthreshold depolarization in the GCs. ***B***, Coactivation of the SuM input (green) and PP input (red). The spike generation in the D-IN (violet action potential) is reinforced by PP stimulation. S-IN is recruited into the network by the PP input (orange action potential). The summation of the synapses results in enhanced EPSP (E)-spike (S) coupling (gray E-S coupling) in the GC. ***C***, Coactivation of SuM and PP inputs during LTP induction; 20 Hz simultaneous activation of SuM (green spikes) and PP (red spikes). During this LTP induction protocol, spike generation in D-IN is strongly reinforced through the entire phase of the stimulation trains, whereas S-IN generates spikes only at the early phase (orange spikes); this could result in a late somatic disinhibition of GC. The synaptic summation during this induction protocol leads to net increase in spike generation in GC (gray spikes). ***D***, Synaptic output by PP activation alone after LTP induction. Both PP-GC synapse and E-S coupling are enhanced.

Here, we proposed a network mechanism by which the SuM input modulates the input-output logic of the DG network (Fig. 9). As shown by our experimental data, SuM neurons corelease glutamate and GABA. According to our study, S-INs receive greater synaptic inhibition than excitation (E < I), whereas D-INs receive stronger synaptic excitation than inhibition (E > I). Moreover, only D-INs generate spikes in response to SuM activation (Fig. 9*A*), whereas S-INs respond with biphasic subthreshold potential changes (fast EPSP and slow IPSP). Our previous studies demonstrated that single action potential generation in D-INs hardly triggers synaptic release onto GCs (Liu et al., 2014) and is therefore ineffective in modulating the GC output (Lee et al., 2016). Thus, SuM activation alone primarily causes small excitatory (red) and large inhibitory (blue) conductance changes around the somata of GCs (Fig. 9*A*). As shown by our previous study (Chiang et al., 2012), GABA is depolarizing as the E_GABA_ (∼−72 mV) > resting membrane potential in GCs and could promote spike generation in GCs in response to the cortical input. The summation of the glutamate- and GABA-mediated conductances therefore results in subthreshold postsynaptic depolarization in GCs (Fig. 9*A*). In great contrast to the SuM input, the PP input alone is sufficient to evoke spikes in S-INs (Liu et al., 2014; Lee et al., 2016). Accordingly, we propose that coactivation of SuM and PP inputs can trigger action potentials in both D-INs and S-INs (Fig. 9*B*). Of note, D-INs and S-INs form reciprocal inhibition (Scharfman et al., 1990; Sik et al., 1997; Liu et al., 2014; Savanthrapadian et al., 2014). Thus, activation of the PP, SuM, and S-INs results in monosynaptic glutamatergic, monosynaptic glutamatergic-GABAergic, and disynaptic somatic GABAergic conductance changes in GCs, respectively (Fig. 9*B*). In line with our experimental data, the synaptic summation of these inputs results in action potential generation in GCs (Fig. 9*B*). During 20 Hz coactivation of the PP and SuM inputs, both D-INs and S-INs generate repetitive spikes (Fig. 9*C*). Notably, D-INs dramatically increase their synaptic output while they fire at burst frequency >20 Hz (Liu et al., 2014). Accordingly, activation of the PP, SuM, S-INs, and D-INs results in monosynaptic glutamatergic, monosynaptic glutamatergic-GABAergic, disynaptic somatic, and disynaptic dendritic GABAergic conductance changes in GCs, respectively (Fig. 9*C*). Overall, the synaptic summation of these inputs at 20 Hz results in multiple action potentials in GCs (Fig. 9*C*), which is supported by our experimental data (Fig. 8*D*). The enhanced spike generation in GCs during LTP induction is believed to be essential during the induction of Hebbian LTP.

After LTP induction, the pSpike was greatly enhanced (Fig. 8*F*), whereas the fEPSP was modestly enhanced (Fig. 8*G*). Although several potential mechanisms could account for these changes, a parsimonious explanation is the formation of Hebbian LTP (Fig. 9*D*). Specifically, activity-dependent Hebbian LTP is accompanied by synaptic potentiation or a long-lasting increase in GC excitability as demonstrated by enhanced EPSP-spike (E-S) coupling (Fig. 8*F*). Alternatively, the enhancement of E-S coupling after LTP induction could be mediated through network mechanisms. Given that the fEPSP at the PP-GC synapse was modestly increased (Fig. 8*G*), we proposed that the D-IN-GC synapse may undergo weak LTD (iLTD), resulting a slight increase in the fEPSP (Fig. 8*G*) after LTP induction. In contrast, the S-IN-GC synapse undergoes strong iLTD, resulting in a large decrease in somatic inhibition and therefore a large increase in the pSpike (Fig. 8*F*). The future work is to investigate the changes in the synaptic efficacy at individual synapse in the DG circuits after LTP induction.

### Cortical and subcortical afferents differentially recruit distinct types of DG INs

Extrinsic inputs differentially activate subtypes of GABAergic INs in the DG and play important roles in gating information transmission to the hippocampus (Hefft and Jonas, 2005; Ewell and Jones, 2010; Armstrong et al., 2011; Chiang et al., 2012; Liu et al., 2014; Hsu et al., 2016; Lee et al., 2016). We recently demonstrated that the commissural fibers of hilar MCs provide a strong excitatory drive to the S-INs, and D-INs, including ML cells and TML cells, while the medial PP provides strong excitatory input to the S-INs (Hsu et al., 2016). In contrast, HIPP and HICAP cells receive weak excitatory inputs from the PP and are weakly recruited by the commissural fibers of hilar MCs (Hsu et al., 2016). This study revealed that activation of the SuM input alone can reliably recruit HIPP and HICAP cells. We have shown that both HIPP and HICAP cells dynamically regulate dendritic excitability of GCs (Liu et al., 2014). They weakly inhibit GCs when they fire sparsely, whereas they inhibit GCs robustly in the burst spiking mode (Liu et al., 2014). Overall, cortical and subcortical inputs may engage in hippocampal-dependent functions, such as cognition and affective behaviors through differential recruitment of distinct types of DG INs.

### SuM input differentially regulate inhibitory circuits

Although INs primarily innervate principal neurons, a growing body of evidence shows that DG INs connect and inhibit each other (Wang and Buzsáki, 1996; Bartos et al., 2007; Liu et al., 2014). Here, we show that the SuM input robustly recruits HIPP, TML, MOPP, and HICAP cells in the DG. These types of D-INs, especially HIPP and HICAP, are known to form synaptic connections with fast-spiking basket cells (BCs) (12.8% connectivity at HIPP-BC synapses and 16.3% connectivity at HICAP-BC synapses) and effectively inhibit spike generation and reduce spike jitters in BCs (Acsády et al., 2000; Savanthrapadian et al., 2014). Therefore, their direct or indirect activation could cause somatic disinhibition in GCs and result in increased GC excitability. The DG ensembles are highly sensitive to the change of contextual cues (Danielson et al., 2016; Pignatelli et al., 2019). Somatostatin-expressing cells, including HIPP and TML cells, control the size of memory ensembles (Stefanelli et al., 2016). Therefore, activation of HIPP cells by the SuM input could regulate the size and specificity of memory engram.

GABAergic INs are believed to generate and maintain hippocampal theta activity (Freund and Buzsáki, 1996; Fricker and Miles, 2001; McBain and Fisahn, 2001; Freund, 2003; Ito et al., 2018). Given that the SuM plays an essential role in the generation and regulation of hippocampal theta activity, it would be interesting to determine the process by which D-INs are selectively recruited by SuM neurons *in vivo*. It will be more physiologically relevant to determine the process by which target cell-specific cotransmission of glutamate and GABA at the SuM-DG synapses contributes to brain computation in different behavioral states. The high excitation/low inhibition (E > I) at the SuM-D-IN synapses can promote dendritic inhibition, whereas the low excitation/high inhibition (E < I) at the SuM-GC synapses may help maintain minimal excitatory drive to GCs on one hand, and ensure high spiking precision on the other hand. The differential cotransmission of these two contrasting neurotransmitters at these two synapses may be crucial to the sparsity of GC activation, which plays a central role in pattern separation.

Correct representation of sensory information relies on the precise temporal firing of neurons (Reich et al., 1997; Kara et al., 2000; Reinagel and Reid, 2002). Here, we demonstrated that SuM-mediated glutamate-GABA cotransmission promotes spike-timing fidelity and reduces action potential latency in GCs. This could be essential for ensuring the temporal precision of cognition and fidelity in separating barrage of sensory information into distinct outputs, as described in pattern separation. Moreover, the interaction among coincident inputs gives rise to associative plasticity and long-term regulation of information flow. Consistent with this view, pairing the SuM input with the PP enhances the responses of GCs to cortical inputs, and also promotes long-lasting increase in the excitability of GCs. During LTP induction (Fig. 8*C*), spikes are reliably generated in GCs. After the LTP induction, the PP-GC synapse is strengthened, and there is a long-lasting increase in the excitability of GCs. In addition to synaptic summation, the observed net enhancement of GCs activity could be explained by IN network functions as illustrated in our proposed models (Fig. 9). Given that fast-spiking BCs in the DG provide powerful inhibition onto GCs, suppression of their activities increases the response of GCs to the cortical input (Lee et al., 2016). Notably, dendritic inhibition driven by HIPP cells can reduce spike generation in BCs (Savanthrapadian et al., 2014). Our study showed that activation of the SuM input reliably excites HIPP and TML cells, which could suppress BCs activities, leading to somatic disinhibition of GCs and enhanced spike generation.
